# Potential of Nanomaterial Applications in Dietary Supplements and Foods for Special Medical Purposes

**DOI:** 10.3390/nano9020296

**Published:** 2019-02-19

**Authors:** Josef Jampilek, Jiri Kos, Katarina Kralova

**Affiliations:** 1 Division of Biologically Active Complexes and Molecular Magnets, Regional Centre of Advanced Technologies and Materials, Faculty of Science, Palacky University, Slechtitelu 27, 783 71 Olomouc, Czech Republic; 2 Institute of Neuroimmunology, Slovak Academy of Sciences, Dubravska cesta 9, 845 10 Bratislava, Slovakia; 3 Department of Pharmaceutical Chemistry, Faculty of Pharmacy, Comenius University, Odbojarov 10, 832 32 Bratislava, Slovakia; jirikos85@gmail.com; 4 Institute of Chemistry, Faculty of Natural Sciences, Comenius University, Ilkovicova 6, 842 15 Bratislava, Slovakia; kata.kralova@gmail.com

**Keywords:** bioactive agents, dietary supplements, foodstuffs, feed, nanoparticles, nanoformulations, nanoemulsions, nutraceuticals, encapsulation

## Abstract

Dietary supplements and foods for special medical purposes are special medical products classified according to the legal basis. They are regulated, for example, by the European Food Safety Authority and the U.S. Food and Drug Administration, as well as by various national regulations issued most frequently by the Ministry of Health and/or the Ministry of Agriculture of particular countries around the world. They constitute a concentrated source of vitamins, minerals, polyunsaturated fatty acids and antioxidants or other compounds with a nutritional or physiological effect contained in the food/feed, alone or in combination, intended for direct consumption in small measured amounts. As nanotechnology provides “a new dimension” accompanied with new or modified properties conferred to many current materials, it is widely used for the production of a new generation of drug formulations, and it is also used in the food industry and even in various types of nutritional supplements. These nanoformulations of supplements are being prepared especially with the purpose to improve bioavailability, protect active ingredients against degradation, or reduce side effects. This contribution comprehensively summarizes the current state of the research focused on nanoformulated human and veterinary dietary supplements, nutraceuticals, and functional foods for special medical purposes, their particular applications in various food products and drinks as well as the most important related guidelines, regulations and directives.

## 1. Introduction

Fortification of edible products (e.g., food, food constituents, or supplements) with nutrients or non-nutrient bioactive components can help to balance the total nutrient profile of a diet and supplement nutrients lost in processing and thus to correct or prevent insufficient nutrient intake and associated deficiencies [1]. Compounds of natural origin, such as curcumin (CUR) occurring in turmeric, ω-3-fatty acid in fish oil, vitamins from fruits, when encapsulated in an appropriate nanocarrier, will be released after consumption of the food in the target organ and utilized according to its nutritional property [2].

Basic types of preparations/materials influencing human health or condition can be classified as follows: (i) drug products, (ii) homeopathics, (iii) dietary supplements (DISs), (iv) medical devices, (v) cosmetics, and (vi) biocidal products. Dietary (food) supplements are products that look similar to medicines (can be sold in pharmacies) but are a special category of foods. They contain vitamins, minerals, amino acids, essential fatty acids, natural products, probiotics, etc., as active ingredients. The purpose of a DIS is to keep the human body functioning properly by delivering compounds that are needed by the human body but could not be received sufficiently from a regular diet. According to the manufacturers, DISs have beneficial effects on health conditions. They are manufactured in the form of pills, capsules, tablets, or liquids [3,4]. DISs are regulated by many guidelines, regulations and directives, for example, by the European Commission directives 2002/46/EC and 2006/37/EC, European regulations 1924/2006, 1137/2008, 1170/2009, 1161/2011, 119/2014, 2015/414, 2017/1203 [4], and by a number of documents published by the U.S. Food and Drug Administration (FDA) [5], to ensure the quality and safety of these products, to protect consumers against potential health risks from such products, and to ensure that they are not provided with misleading information. In addition, DISs are regulated by the European Food Safety Authority (EFSA), national legislation (e.g., on food) and regulations (e.g., requirements for food supplements and food enrichment), etc. For example, in the EU market, approx. 30 approved nutrition claims (meaning that specific requirements are to be met) can be found, and a product can be marketed as a DIS only if it meets the so-called health claims, which is any statement about a relationship between food and health. The European Commission approves various health claims, which have to be easily understood by consumers, based on scientific evidence. The EFSA is responsible for evaluating the scientific evidence supporting health claims, the types of which are as follows: (i) ‘Function Health Claims’ (relating to the growth, development and functions of the body, or referring to psychological and behavioral functions, or on slimming or weight-control), (ii) ‘Risk Reduction Claims’ (on reducing a risk factor in the development of a disease), and (iii) Health ‘Claims referring to children’s development’ [6].

It is important to note that DISs are not food additives, which are special excipients added to foods for modifications of their flavor, color, or longevity [7].

Foods for special medical purposes (FSMPs), based on the definition of the EFSA, “are designed to feed patients who, because of a particular disease, disorder, or medical condition, have nutritional needs that cannot be met by consuming standard foodstuffs. Specifically, according to EU legislation, they are intended for patients with a limited, impaired, or disturbed capacity to take, digest, absorb, metabolize, or excrete ordinary foods, or certain nutrients or metabolites; or with other medically nutrient requirements whose dietary management cannot be achieved by modification of the normal diet alone” [8]. It means that FSMPs are foods that are intended for nourishment at: (i) certain groups of people whose digestive process or metabolism is impaired, (ii) certain groups of persons in a particular physiological state, which therefore, may have specific benefits from controlled consumption of certain substances in food, or (iii) healthy infants and young children. Therefore, the following categories of FSMPs can be distinguished: (i) food for infant and follow-on nourishment and nutrition of small children, (ii) food for cereal and other non-cereal food for infant and young children, (iii) low-energy foods designed to reduce body weight, (iv) food without phenylalanine, (v) gluten-free foods, (vi) foods for people with disorders of carbohydrate metabolism (diabetics), (vii) low lactose or lactose-free foods, (viii) foods with low protein content, (ix) foods intended for athletes and for persons with increased physical performance. FSMPs are advised to be used only under medical supervision and have to be provided with labels with information about their intended use. The European Commission also issued several documents for the regulation of FSMPs, e.g., Commission Directive 1999/21/EC, Commission Regulations No. 953/2009, 609/2013, 2016/128 [9], and so-called “medical foods” are regulated also by FDA [10].

A functional food or functional ingredient is any food or food component providing health benefits beyond basic nutrition, and natural bioactive compounds as functional ingredients showing beneficial effects for health become increasingly popular in the diet [11]. Therefore, functional foods are similar to traditional conventional foods but have more advantageous properties in relation to healthy physical condition. Nutraceuticals are based on both food and herbal or other natural products and are used in the form of pharmaceutical formulations, i.e., tablets, capsules, drops, or liquids, and have physiological benefits. The main focus of all these products is to improve health and reduce the risk of disease. In contrast to drugs, in all these cases, the active substance or a mixture of active compounds is present in low concentration [12].

Nanotechnology is a rapidly growing field that ensures the development of materials with new dimensions, novel properties, and a wider range of applications. U.S. National Nanotechnology Initiative defines nanoparticles (NPs) in the range of 1–100 nm [13]. According to the Recommendation on the definition of a nanomaterial adopted by the European Commission, the term “nanomaterial” means “a natural, incidental or manufactured material containing particles, in an unbound state or as an aggregate or as an agglomerate and where, for 50% or more of the particles in the number size distribution, one or more external dimensions is in the size range 1–100 nm. In specific cases and where warranted by concerns for the environment, health, safety or competitiveness the number size distribution threshold of 50% may be replaced by a threshold between 1 and 50%” [14]. However, in pharmacy, particles of 10–500 nm have been used, rarely up to 700 nm. From the aspect of passage through vessels, the inside diameter of which is in the range from 25 mm (aorta) to 5 μm (capillaries), the ideal size of NPs should be <300 nm to ensure efficient transport for targeted distribution of drugs [15,16,17,18,19,20].

NPs can be prepared from both inorganic and organic materials [21,22,23], and currently, especially encapsulation to various biodegradable nature-based biopolymers is more and more frequently used [19,20,24,25]. NPs can be generated by either top-down methods (dispergation, fluidization, homogenization processes, or emulsifying technologies) or bottom-up methods (precipitation/condensation processes, evaporation techniques, various controlled sol-gel syntheses) [21,26]. NPs produced using mechanical approaches are usually, in the range 100–1000 nm; to produce NPs of size 10–100 nm, chemical and bottom-up methods are used [21,27]. “Green” synthesis of NPs or innovative biotechnological approaches related to the synthesis of NPs are summarized by Singh and Shukla et al. [22,23].

The physical, chemical, and biological properties of nanoscale materials are significantly, different from those of bulk materials and single atoms or molecules; therefore, different properties of active pharmaceutical ingredients have been modified in such a way [14,28,29,30,31,32,33]. In biomedical branches, NPs can be used for nanodiagnostics, as nanomaterials for tissue engineering, as drug carriers for specific delivery/targeted biodistribution or controlled release, and as agents/drugs for prevention/treatment of diseases. Therefore, application of nanotechnology can be considered as an excellent tool for modification of parameters of bioactive agents. Modification of properties using nanosystems/nanoformulations helps to enhance the bioavailability of active substances and change the route of administration when needed. Therefore, smaller amounts of substances can be used, which allows decreasing dose-dependent toxicity and various side effects. In addition, many formulations also protect bioactive molecules from degradation [19,20,24,25,28,29,31,34,35,36,37,38,39,40,41,42]. The enhancement of bioavailability could be achieved by the improved solubility of bioactive compounds under gastrointestinal (GI) conditions, their protection from the chemical conditions in the GI tract, and controlled release within the GI tract, or by an improved transfer through the intestinal wall, and the particle size, surface properties, and physical state of the nanomaterials used in food supplements are crucial characteristics affecting their final nutritional value [43]. Recent findings and advancements related to lipid nanoscale cargos for the protection and delivery of food bioactive ingredients and nutraceuticals were overviewed by Akhavan et al. [44]. Nanoemulsion (NE) compositions, types of active ingredients, applications in different types of food systems, toxicological and safety aspects, and future directions were summarized by Kumar and Sarkar [45]. For encapsulating drugs/nutraceuticals and fortification of food products, especially beverages with water insoluble nutraceuticals, nanostructured lipid carriers (NLCs) could be successfully applied [46]. Micro- and nano bio-based delivery systems (DESs) for food applications were discussed also by Simoes et al. [47].

The above-mentioned nanoformulations can be found in many drug classes, and so it is not surprising that supplements and FSMPs have also started being formulated in the nanoscale, especially with the aim to improve bioavailability, protect active ingredients against degradation, or reduce side effects. Therefore, this contribution summarizes the current state of the research focused on nanoformulated human and veterinary DISs and FSMPs.

## 2. Types of Formulations and Used Materials

**Figure 1:**
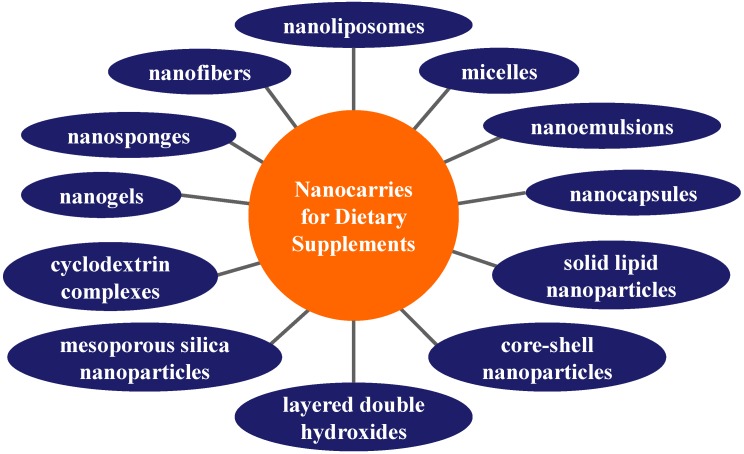
Most frequently used nanoformulation types of dietary supplements and foods for special medical purposes.

Nutraceuticals’ functionality in food products can be stabilized and enhanced using bio-based nanoscaled DESs that help to improve their bioavailability and protect valuable nutraceuticals at food processing or digestion, see Figure 1, where individual most frequently applied nanoformulations are mentioned. Selected nanoformulations are discussed below in the following subchapters. Advances in nutraceutical DESs with focus on the formulation design for the enhancement of nutraceuticals’ bioavailability with the purpose to ensure effective preservation or maximization of their bioactivity and safety inside the human body were summarized by Goncalves et al. [48]. A review of recent research developments related to nanocarrier-based delivery of nutraceuticals for cancer prevention and treatment was presented by Arora and Jaglan [49]. Recent findings related to advances made in the nanoencapsulation of lipophilic and hydrophilic vitamins, safety issues, and health risks regarding the consumption of these products, which would result in widespread utilization of nanoencapsulated vitamins in the food and beverage products in the future, were summarized by Katouzian and Jafari [50]. The intelligent DESs for bioactive compounds in foods designed to improve their low solubility, poor stability, and low permeability in the GI tract and improving their oral bioavailability were discussed by Chai et al. [51] from the aspect of physicochemical and physiological conditions, absorption mechanisms, obstacles, and responsive strategies.

Gleeson et al. [52] looked into the potential of certain delivery strategies for the improvement of the oral bioavailability of different types of nutraceuticals, such as fatty acids, bioactive peptides, micronutrients, and phytochemicals, and emphasized that nutraceutical and pharmaceutical industries could leverage approaches to oral delivery formulations, which would result in synergies for nutraceutical and pharmaceutical molecules. For example, microfluidization could be considered as an efficient emulsification technique resulting in fish oil encapsulated powder producing emulsions at the nanoscale range (*d*_43_ of 210–280 nm) with the lowest unencapsulated oil at the surface of particles [53]. At investigating the effect of excipient emulsions with different surface-weighted mean droplet diameters *d*_32_ = 0.15 μm (small), 0.40 μm (medium), and 22.3 μm (large) on the bioaccessibility of carotenoids from tomatoes using a simulated GI tract, it was found that the bioaccessibility of carotenoids decreased with an increase of initial droplet size, which could be attributed to more efficient extraction of carotenoids from tomato tissues by smaller droplets that were digested faster. This caused faster mixed micelle formation and, consequently, enhanced solubilization of carotenoids in intestinal fluids. Moreover, when tomatoes were boiled with emulsions, the bioaccessibility of carotenoids was higher than when they were boiled alone and subsequently added to emulsions [54]. Electrospinning and electrospraying technologies constitute useful and modern techniques used for the encapsulation and controlled release of bioactive compounds, including drugs and health-promoting agents. Both electrospinning, mostly used for fibres, and electrospraying, mostly used for particles, are voltage-driven fabrication technologies enabling tight control of fibres and particles in the micro-, submicro- and nanoscale dimensions suitable for a wide range (polymers, proteins, inorganic) of materials. Both processes are able to replace traditional techniques, e.g., spray-drying or lyophilisation, as they propose several benefits such as (i) production of dry products in a single step, (ii) room temperature operation (suitable for labile components, e.g., antioxidants, omega-3 oils, living cells, etc.), (iii) enabling to produce single-phase or multi-component fibres and particles, and (iv) high effectivity of encapsulation [53,55,56,57,58,59,60], as mentioned below.

### 2.1. Liposomes and Nanoscale Emulsions

Nanoliposomes, or nanometric bilayer phospholipid vesicles, have a very promising potential for the nutraceutical industry, because they can encapsulate simultaneously lipophilic and hydrophilic materials, ensuring a synergistic effect, and can protect sensitive bioactive compounds, enhance their bioavailability, ensure sustained-release, and improve storage stability. The unique properties of nanoliposomes predestine them to be used in DISs for effective disease prevention and health promotion [61].

Nanophytosome is one of the newest lipid-based nanocarriers enabling the delivery of botanical based nutraceuticals, which could be potentially used in food products for designing novel functional foods and beverages [62]. Phytosomes-phosphatidylcholine (PC)—rutin complexes prepared by the encapsulation of rutin with PC using rutin:PC molar ratio 1:3 were found to provide the highest physical and chemical stability (during 30 days of storage) with fine particle sizes (<100 nm) and the encapsulation efficiency (EE) of 99%, and due to the ability of masking undesirable features of rutin, they may be applied in fortification of food products with water insoluble nutraceuticals [63].

At the preparation of liposomes starting from multilamellar large vesicles with a diameter range 2.9–5.7 μm using an ultrasound-assisted approach based on the thin-film hydration method, unilamellar vesicles with diameter sizes ranging from 40 nm to 51 nm were achieved, showing the EE of 56% for cobalamin, 76% for α-tocopherol, and 57% for ergocalciferol. The nanovesicles and their content were kept intact for >10 days when incubated at simulated conditions of extracellular environment thanks to the used lipid composition [64]. The investigation of the encapsulation and preservation of quercetin (Q) with cyclodextrins (CDs), conventional liposomes composed of three different types of phospholipids (unsaturated egg Lipoid E80, unsaturated soybean Lipoid S100, and saturated soybean Phospholipon 90H), and drug-in-CD-in-liposomes showed that the application of Lipoid E80-liposomes resulted in a better protection of Q against UV irradiation, and its photostability was additionally improved when encapsulated in drug-in-CD-in-liposomes (sulfobutylether β-CD/Q inclusion complex in Lipoid E80 liposomes) [65].

Compared to CUR liposomes, the Pluronic^®^ modified CUR liposomes showed a slower release rate and lower cumulative release percentage for CUR, enhanced pH stability and thermal stability, and pronouncedly improved absorption in simulated GI tract in vitro, suggesting that both types of liposomes could be used as carriers of CUR in nutraceuticals and functional foods. The best bioaccessibility was observed for CUR liposomes modified with Pluronic^®^ F-127 [66].

Stimuli-sensitive (smart) nano DESs for nutraceuticals of both a nutritional and pharmaceutical value are of great importance for the formulation of novel functional foods. Because the best effect on the human health was observed when the weight ratio of ω-6/ω-3 polyunsaturated fatty acids (PUFAs) is in the range between 1:1 and 5:1, Semenova et al. [67] focused their attention on the molecular design of DESs on the basis of nanoscale complexes formed between a covalent conjugate (sodium caseinate (SCas) + maltodextrin; dextrose equivalent = 2) and combinations of polyunsaturated lipids that are mutually complementary in the content of ω-6 and ω-3 PUFAs: α-linolenic acid (α-LNA) + α-linoleic acid (α-LLA); liposomes of soy PC + α-LNA, and micelles of soy lyso-PC + α-LNA. The researchers concluded that thanks to the EE of all these lipid combinations by the conjugate, lipids were highly protected against oxidation, and their high solubility in an aqueous medium was reached. Dey et al. [68] designed ω-3 PUFA enriched biocompatible NE with sesame protein isolate (SPI) as a natural surfactant. NE with 0.5% (*w*/*v*) SPI and Tween 20 and Span 80 used in 1:1 ratio having the hydrodynamic droplet size of 89.68 ± 2.38 nm effectively enhanced the shelf-life stability of NEs, and the fatty acid release from NE droplets was ≥90% during 120 min of simulated two-step in vitro digestion.

The short-chain triglyceride-based NE encapsulating vitamin E did not physically withstand temperatures exceeding 25 °C, while with long-chain triglyceride-based NEs, good vitamin E retention even at 40 °C was observed, and the retention was increased when the NEs were stored in the dark [69]. Vitamin D NEs with small droplet diameters (*d* < 200 nm) fabricated by spontaneous emulsification using medium chain triglycerides (MCT) and Tween 80 at surfactant-to-oil ratio ≥1 at high stirring speeds (800 rpm) were found to be relatively stable at ambient temperatures and unstable at heating (T > 80 °C), but the application of a cosurfactant (sodium dodecyl sulfate) could improve their thermal stability [70]. The investigation of the effect of excipient NEs formulated from long or medium chain triglycerides (LCT or MCT) on β-carotene (β-Car) bioaccessibility from commercial DISs (tablets or soft gels) studied using an in vitro GI tract model showed that the application of LCT NEs enhanced β-Car bioaccessibility from tablets and soft gels by 20% and 5%, respectively, while the effect of MCTs was minor. This could be connected with the fact that large carotenoid molecules could be incorporated only into large mixed micelles formed by LCT digestion, and thus, excipient NEs could be applied to improve nutraceutical bioavailability from DISs [71]. NEs prepared using three LCT oils (flaxseed, olive and corn oil) increased the bioaccessibility of astaxanthin (AST) compared to the control due to the formation of mixed micelles that solubilized the hydrophobic carotenoids. The final amount of free fatty acids released affected lipid digestion and AST bioaccessibility, which decreased in the following order: olive oil > flaxseed oil > corn oil, and free fatty acids unsaturation and chain length affected lipid digestion and micelle formation [72]. Saxena et al. [73] increased the bioavailability of the model bioactive compound α-tocopherol as a food supplement using edible (coconut) oil NEs. The prepared NEs were found stable and biocompatible, and the contribution of kinetic-controlled release was found to be approx. 70%, while that of diffusion-controlled release was approx. 30%, suggesting the potential of the use of edible oil NEs in food and beverages.

A saponin coated NE with mean droplet diameter 277 nm encapsulating vitamin E was found to be more stable to droplet coalescence at thermal processing (30–90 °C), long-term storage, and mechanical stress than a conventional emulsion with mean droplet diameter 1.285 μm. At application of both emulsion formulations to male Wistar rats, droplet flocculation and coalescence during in vivo digestion was observed, however, the higher in vivo oral bioavailability of vitamin E encapsulated in the NE was reflected in a 3-fold increase in the area under the curve (AUC) compared to the conventional emulsion [74]. The lowest particle diameters (*d*_32_) of vitamin E NEs fabricated using natural surfactants, quillaja saponin, and lecithin and high-pressure homogenization were 0.13 μm for lecithin and 0.12 μm for quillaja saponin at vitamin E to orange oil ratio 50:50%. At pH 7, both systems were stable in the temperature range 3–90 °C but unstable at pH 2 or in the presence of NaCl (>100 mM NaCl for lecithin and ≥400 mM NaCl for quillaja saponin) [75]. The encapsulation of CUR in saponin-coated CUR NEs fabricated using a simple pH-driven loading method improved CUR solubility and bioavailability, and its in vitro bioaccessibility was approx. 3.3-fold higher compared to free CUR. In an in vivo study, oral administration of these NPs to Sprague Dawley rats resulted in approx. 8.9-fold higher in vivo bioavailability than that estimated with free CUR [76].

Zheng et al. [77] subjected CUR loaded oil-in-water (O/W) NEs prepared using the conventional oil-loading method, the heat-driven method, and the pH-driven method and three commercial CUR supplements (Nature Made, Full Spectrum, and CurcuWin) to a simulated GI tract model consisting of mouth, stomach, and small intestine phases and found that the three tested NEs showed similar CUR bioaccessibility (74–79%) with the highest absolute amount of CUR in the mixed micelle phase of the NE fabricated by the pH-driven method. The concentration of CUR in mixed micelles decreased as follows: CurcuWin ≈ pH-driven method > heat-driven method > conventional method >> full spectrum > nature made, and CUR encapsulated in small lipid particles had an improved absorption in GI tract.

Cholecalciferol (vitamin D_3_) minitablets and an optimized bile salt/lipase alginate-glycerin film provided unique oral components for inclusion in a bioactive association platform (BAP) capsule designed to deliver the active nutraceutical ingredient from the formulation framework resulting in the enhanced in vitro and in vivo performance of cholecalciferol. The in vivo experiment showed that cholecalciferol bioavailability from the BAP was 3.2-fold greater than that of the conventional product, and improved and maintained serum levels of 25-hydroxyvitamin D_3_ were observed as well, suggesting that BAP could be considered as an ideal oral vehicle for enhanced delivery of cholecalciferol [78].

β-Car enriched O/W emulsions, in which chlorogenic acid-lactoferrin-polydextrose conjugate was used as an emulsifier to stabilize lipid droplets, showed improved stability to droplet aggregation under simulated GI tract conditions, resulting in increased β-Car bioaccessibility, suggesting that the ternary conjugate-stabilized emulsions could be used as protectors and carriers of hydrophobic drugs, supplements, and nutraceuticals [79]. On the other hand, excipient NEs had much less effect on the bioaccessibility of phenolic compounds, probably due to their smaller and more polar molecules, which could be more easily solubilized in aqueous intestinal fluids [80].

Among NEs prepared using soy protein isolate (132 nm), whey protein concentrate (190 nm), maltodextrin (266 nm), and gum arabic (468 nm), the soy protein isolate NE showing the smallest droplet size provided the highest protection of vitamin D (85%) at 4 wt % concentration, pH 7, and 25 °C [81].

### 2.2. Lipid-Based Carriers

Nanocapsules based on lipid formulations having larger surface area than microsized carriers can more effectively enhance solubility, bioavailability, and controlled release of nanoencapsulated phenolic compounds and could be successfully applied in functional foods [82]. For example, the physical stability of β-Car nanocapsules (>300 nm) showed only minor changes during storage, suggesting that they could be used in functional beverages and foods as well as nutraceutical products [83]. At the investigation of the impact of solid domain properties on the rate of compound release from NLCs using Monte Carlo simulations, it was found that the release of encapsulated bioactive compounds by solid impenetrable domains at the particle/solution interface is hindered only when the domain size is much smaller than the size of NPs, even if a considerable proportion of the interface is covered by these domains, with the rate of release depending also on the geometry of the solid domains [84]. The preparation and characterization of vitamin A palmitate-loaded NLCs as DESs for food products was reported by Kong et al. [85].

Solid lipid microparticles (MPs) loaded with 0.1% of vitamin D_3_ by spray chilling with mean diameter 83.0–98.6 μm that were fabricated using vegetable fat as a carrier and beeswax (1% of the formulation) showed increased vitamin stability at 25 °C, and 86.3% of vitamin were detected after 65 days of storage compared to 60.8% estimated with non-immobilized vitamin at same conditions, suggesting the potential of using such formulation in foods [86].

Prolonged physical stability at room and refrigerated temperature conditions as well as an increase in the bioavailability of encapsulated CUR compared to that of CUR suspensions was shown by chitosan (CS) coated solid lipid NPs incorporating this nutraceutical after oral administration [87].

In a systematic review, Nunes et al. [88] focused on the use of solid lipid NPs as oral DESs of phenolic compounds that allow overcoming the pharmacokinetic limitations of these compounds and ameliorate their nutraceutical potential.

### 2.3. Polysaccharide Matrices

Polysaccharides that have various enzymatic susceptibilities to ensure specific degradation in the small or large intestine when used as a NP coating can efficiently retard the nonspecific release of encapsulated bioactive compounds until the coating is exposed to its intended environment of release, and such coated NPs can be potentially targeted to different GI tract organs and taken up by the enterocytes, providing improved oral bioavailability [89].

High amylose corn and potato starches nanocarriers with granular structure and particle sizes ranging from 32.04 to 99.2 nm were used to encapsulate vitamin D_3_, and their EE ranged from 22.34 to 94.8%. By using ultrasonic treatment, an increase of the hydrocarbon chain length was observed resulting in van der Waals and *H*-bonds of vitamin D_3_ with the potato starch and greater thermal stability [90]. Low-molecular-weight octenyl succinic anhydride modified starches were reported to be suitable to form stable vitamin E nanocapsules for potential application in beverages [91].

Recent findings concerning the use of cellulosic nanomaterials for food and nutraceutical needs were summarized by Khan et al. [92]. The addition of cellulose nanocrystals and lecithin into alginate microbeads improved the viability of encapsulated probiotic (*Lactobacillus rhamnosus* ATCC 9595) during gastric passage and storage, and at 25 and 4 °C storage conditions, a decrease in the viability of *L. rhamnosus* by 1.23 and 1.08 log, respectively, was estimated, while at encapsulation of the probiotic with alginate microbeads, a 3.17 and 1.93 log reduction, respectively, was observed [93]. The oligo-hyalurosomes nanoscale DES based on oligo-hyaluronic acid-CUR polymer co-loaded with both CUR and resveratrol (RES) showing the average particle size of 134.5 ± 5.1 nm, spherical shape, and zeta potential of −29.4 ± 1.2 mV at pH 7.4 phosphate buffer conditions exhibited excellent stability and sustained release character and higher radical scavenging activity compared to the single formulations and liposomes suggesting that this system could be considered as a promising nanofood DES applicable in juice, yoghourt and nutritional supplements [94].

CS/tripolyphosphate-nanoliposomes core-shell nanocomplexes as vitamin E carriers showed vitamin E retention rate >80% during the 30-day storage and 92% and 97% after heating at 65 °C for 30 min and at 80 °C for 16 s, respectively, and based on the enhanced stability of liposomes against temperature stress reflected in reduced particle aggregation, zeta potential inversion, and membrane fluidity, this formulation could be considered as appropriate for commercial use in the food industry [95]. CS hydrochloride/carboxymethyl CS nanocomplexes loaded with anthocyanins with particle size 178.1 nm, zeta potential +25.6 mV, and polydispersity index 0.315 showed a higher stability when placed at different conventional storage temperatures, various l-ascorbic acid concentrations, varying pH, or white fluorescent light, suggesting that such nanocomplexes could be applied in food ingredients associated with stable anthocyanins in functional foods and nutraceutical applications [96].

In food-grade alginate/CS nanolaminates obtained by the layer-by-layer technique, in which folic acid (FA) was incorporated by post-diffusion, a higher stability of FA under ultraviolet light exposure compared to free FA was estimated, and the higher rate and concentration of FA released from nanolaminates at pH 7 in comparison with that at pH 3 suggested that nanolaminates containing hydrophilic active compounds can be used for food applications [97].

Insulin encapsulated in antacid-loaded calcium alginate microgels (diameter 280 μm) had higher biological activity in simulated gastric conditions than free insulin, and considerably increased Akt phosphorylation at Thr308 and Ser473 in L6 myotubes was observed [98].

Papagiannopoulos and Vlassi [99] reported preparation of multi-functional stimuli-responsive NPs for food and biomedical applications by combining electrostatic complexation between proteins and polysaccharides with following thermal protein denaturation for the production of chondroitin sulfate/bovine serum albumin NPs. The irreversible protein–protein contacts upon temperature treatment provide the complexes with properties of nanogels, and the surface charge of the prepared NPs reversed at pH 5.3, while their size depended on the solution ionic strength and pH. Protein-polysaccharide-surfactant ternary complex particles prepared by anti-solvent co-precipitation using zein, propylene glycol alginate, and either rhamnolipid or lecithin pronouncedly improved the photostability and bioaccessibility of CUR suggesting that they could be used to deliver hydrophobic nutraceuticals for applications in foods, supplements, and pharmaceuticals [100].

### 2.4. Protein-Based Carriers

The state of the art of protein-based nanoencapsulation approaches as well as protein modification approaches in order to extend their functionality in nanocarrier systems to achieve an improvement in encapsulation, retention, protection, and release of bioactive agents was summarized by Fathi et al. [101]. A review paper discussing the latest findings concerning the nanoscale phenomena of whey protein denaturation and aggregation, which could contribute to the design of protein nanostructures with new or improved properties for the incorporation of nutraceuticals in food matrices and their release was presented by Ramos et al. [102]. Using whey protein isolate as an encapsulating agent, Parthasarathi and Anandharamakrishnan [103] presented a spray freeze-drying based microencapsulation technique as a promising strategy to enhance the oral bioavailability of poorly water-soluble bioactive compounds like vitamin E.

Significant aggregation and sedimentation of zein NPs encapsulating lutein (ZLNPs) with hydrodynamic radius approx. 75 nm were observed at gastric digestion conditions, and the ZLNPs that were not fully digested by gastric enzymes adhered to lipid droplets; however, the aggregation was reduced and digestion was stimulated when salt (i.e., high ion concentration) was left out. On the other hand, thanks to the encapsulation of lutein into NPs, its digestive stability was increased [104].

The size of egg albumin (Alb)-FA nanocomplexes prepared by mixing egg Alb NPs with FA did not change after adjusting the pH from 3 to 4, but showed considerable increase after adjusting pH to 5, 6, or 7; however, the bioavailability of FA in the form of digested nanocomplexes for *Lactobacillus rhamnosus* was improved [105].

The degree of FA binding to β-lactoglobulin (β-Lglb) and type A gelatin carriers was affected by their pH-dependent zeta-potential, which indicated the occurrence of ionic bonds, and the binding of FA reached 100% at pH 3. At pH 3, particle size considerably increased at increasing the molar FA/protein ratio; however, shifting back the pH to 7 totally reversed it, which means that these formulations could protect FA at pH 3 prevailing in the stomach, but they are strongly favorable for its delivery to the duodenum (pH 7) [106]. β-Lglb nanostructures were reported to be suitable carriers for riboflavin and its controlled release in an in vitro GI system: approx. 11% was released during their passage through the stomach, while 35%, 38%, and 5% of the total riboflavin were released during their passage through duodenum, jejunum, and ileum, respectively. At food simulant conditions (yoghurt simulant, 3% acetic acid), β-Lglb nanostructures were stable for more than 14 days and had protective impact on riboflavin activity, releasing it in a 7-day period [107].

Isolated 7S and 11S globulins (Glbs) obtained from defeated soy flour, which were complexed with FA and included in culture media, showed higher bacterial growth of *Lactobacillus casei* BL23. Therefore, Glbs-FA based nanocomplexes have potential to be used in nutraceutical, pharmaceutical, and food industries [108]. *Lactobacillus casei* BL23 produces microvesicles carrying proteins that have been connected with its probiotic effect, and, using a proteomic approach, Rubio et al. [109] identified proteins described as mediators of *Lactobacillus*’ probiotic effects, namely p40, p75, and the product of LCABL_31160, which was annotated as an adhesion protein. The expression and subsequent encapsulation of proteins into microvesicles of bacteria generally considered as safe could be also used in applications of foods and nutraceuticals.

Negatively charged (−41 mV) sophorolipid-coated CUR NPs with the particle size of 61 nm showing relatively high EE and loading capacity for CUR that was present in an amorphous state exhibited 2.7–3.6-fold higher bioavailability than free CUR crystals, which was connected primarily with their higher bioaccessibility [110].

Protein–lipid composite NPs having a three-layered structure (barley protein layer, α-tocopherol layer, and phospholipid layer) and an inner aqueous compartment to load the hydrophilic nutraceutical vitamin B_12_ exhibited controlled release behavior in simulated GI media, and in an in vivo experiment, the NPs loaded with vitamin B_12_ increased serum vitamin B_12_ levels in rats upon their oral administration and reduced the level of methylmalonic acid more efficiently than the free vitamin B_12_ form without any toxicity of the formulation observed during 14 days. These NPs could be used for increasing vitamin B_12_ absorption upon oral administration [111].

The enhanced physicochemical stability and in vitro bioaccessibility of vitamin D_3_ in corn protein hydrolysate-based vitamin D_3_ nanocomplexes showing spherical structure with sizes 102–121 nm was reported by Lin et al. [112]. In vitamin d–potato protein co-assemblies, the nanocomplexation provided pronounced protection and reduced vitamin D losses during pasteurization and also under several different sets of storage conditions, suggesting that potato protein could be used as a protective carrier for hydrophobic nutraceuticals suitable for enrichment of clear beverages and other food or drink products with beneficial impact on human health [113].

After drying and reconstitution, vitamin D-loaded re-assembled casein micelles (r-CMs) were found to improve the in vitro bioavailability of vitamin D in a Caco-2 cell model and showed strong protective effect against its gastric degradation, providing 4-fold higher bioavailability compared to free vitamin D [114]. Ghayour et al. [115] encapsulated CUR and Q using a hierarchical approach (binding of ligand to SCas with subsequent re-assembling of micellar nanostructures or formation of casein NPs). r-CMs had smaller mean particle size than casein NPs, and the entrapment efficiency of both ligands was >90%. An incorporated phenolic compound showed notably improved chemical stability during an accelerated shelf-life test. The aqueous solubility of CUR and Q after loading in r-CMs was higher than that of free polyphenol molecules, and the viability of treated MCF-7 human breast cancer cells decreased as follows: free polyphenol molecules >> non-digested polyphenol-loaded carriers > digested polyphenol-loaded r-CMs. Based on the investigation of the stability and bioavailability of CUR in mixed SCas and pea protein isolate NEs, Yerramilli et al. [116] reported that pea proteins could be used to partially replace SCas as an emulsion stabilizer for the protection and delivery of oil-soluble bioactive compounds. Based on in vitro proteolysis, it was found that in low-fat yogurt supplemented with the spray- and freeze-dried casein micelles loaded with vitamin D_2_, 90% of the vitamin remained active compared to 67% estimated with free vitamin [117].

FA–loaded casein NPs of 150 nm fabricated with the use of a coacervation process, stabilized with lysine or arginine, and finally dried by spray-drying were administered to laboratory animals p.o. at dose 1 mg FA/kg and ensured considerably higher serum levels of the vitamin than an aqueous solution of FA administered to animals, and the release profile and oral bioavailability of FA were not affected by the treatment of casein NPs by high hydrostatic pressure [118].

### 2.5. Inorganic Matrices

Inorganic porous materials, such as various silica- or aluminosilicate-based materials/composites, clays, calcium carbonate, calcium phosphate, layered double hydroxides (LDHs), etc., have become good candidates for the delivery of a range of drugs, providing some advantages in formulation and engineering. They have suitable architecture, large surface area, and stability in biological fluids; thus, they are used for high loading capacity, controllable release, and improved targeting [119,120,121].

Comparison of four different capped SiO_2_ mesoporous particles (MSPs) (i.e., hollow silica shells, MCM-41, SBA-15 and UVM-7) showed that they were able to hinder the delivery of FA at low pH (to stomach) and deliver large amounts of the vitamin at neutral pH (to intestine); nevertheless, the usage of supports with large pore entrance ensured an initial fast release, while the mesoporous material MCM-41 demonstrated a sustained release over the time [122]. The amine-capped MSPs also hindered the release of FA in gastric fluids (pH 2) and progressively delivered it in the presence of a simulated intestinal juice (pH 7.5) [123]. Similarly, the in vitro digestion procedure showed that mesoporous silica support loaded with FA and functionalized with amines inhibited the release of FA in acidic solution at pH 2 (stomach) and enabled its controlled release in neutral pH (intestine), thereby modulating the bioaccessibility [124]. Ruiz-Rico et al. [125] investigated controlled FA delivery and stability in fruit juices to reduce potential for over-fortification risks by using dated MSPs and observed that the encapsulation of FA into MSPs resulted in considerably improved vitamin stability and contributed to controlled release after consumption by modifying FA bioaccessibility.

RES encapsulated in mesoporous silica (MCM-48) NPs with the particle size of 90 nm did not alter its bioactivity and, at lower concentration, i.e., 5 µg/mL, exhibited higher anti-inflammatory activity compared to RES suspension or its solution [126]. Similar findings concerning the enhancement of the biological activity of RES by colloidal mesoporous silica NPs were reported also by Summerlin et al. [127]. Singh et al. [128] reviewed causes and consequences of micronutrient deficiencies and the bioavailability of nutrients, vitamins, minerals, and silica for food and outlined that the release of nutrients from silica in simulated intestinal fluid is better than in simulated gastric fluid.

Simple powders and tablets of inorganic–organic nanostructured hybrids prepared by intercalating FA in the MgAl-LDH and ZnAl-LDH exhibited enhanced FA release compared to crystalline FA, suggesting that such hybrids could be used to enhance the active ingredient dissolution at low pH values in effective nutraceutical products [129].

The analysis and speciation of selenium in nutritional supplements based on next-generation Se ingredients, i.e., Se forms with lower toxicity, higher bioavailability, and controlled release, such as selenium NPs (SeNPs) and selenized polysaccharides, was presented by Constantinescu-Aruxandei et al. [130].

## 3. Antioxidants

Reactive oxygen species (ROS) is a term used for oxygen containing free radicals (such as O_2_^•^, HO^•^, HO_2_^•^, RO^•^, ROO^•^) or reactive oxygen-containing compounds (such as H_2_O_2_, O_3_, ^1^O_2_), depending on their reactivity and oxidizing ability. ROS participate in diverse chemical reactions (oxidative stress) resulting in the decomposition of biologically active compounds or biomolecules. Antioxidants that are able to protect other molecules from the damaging effects of such ROS can be used as excipients in formulations or as biologically active compounds preventing oxidative stress [131]. Bioactive compounds like polyphenols, flavonoids, and vitamins showing antioxidant properties are suitable to be used for the fortification of food products to enhance their functionality, and therefore, encapsulation systems for the delivery of such nutraceuticals are necessary to overcome their low stability and bioavailability [132]. The choice of the appropriate encapsulation method is essential, because the modification of bioactivity (increase, preservation, or decrease) is affected by interactions established between the functional groups of the encapsulated compound and the encapsulating nanomaterial [133].

### 3.1. Nanoformulations with Antioxidant Capacity

A significant property of nanoformulations is the possibility to co-encapsulate antioxidants together with an active ingredient and thus increase the stability and extend expiration. As antioxidant excipients, PUFAs, carotenoids, antioxidant plant extracts, CUR, and catechins can be used, and as formulations, nano/micro emulsions, NLCs, NPs/MPs, and liposomes can be applied.

The antioxidant capacity of α-LNA loaded microemulsion was strongly enhanced after the introduction of carbon dots, which were distributed mainly at the oil-water interface, suggesting a “turn off” effect of the interface [134]. Benzylisothiocyanate nutraceutical encapsulated in a stable α-tocopherol-based O/W NE stabilized with a nontoxic, biodegradable surfactant, sodium stearoyl lactate, showed better antioxidant activity than pure and CUR encapsulated NEs, however CUR entrapped in the NE was effectively protected from UV light-induced degradation [135].

AST-loaded NLCs with the Z-average size of 94 nm containing α-tocopherol and EDTA as antioxidants that were stabilized using Tween 80 and lecithin and mixed with non-pasteurized CO_2_-free beer at the volume ratio of 3:97 showed improved stability at low storage temperature of 6 °C [136].

Anionic sphere-shaped core-shell NPs with zein-epigallocatechin gallate (EGCG) conjugates as the hydrophobic core and a biosurfactant (rhamnolipid) as a shell with average diameters <200 nm co-loaded with CUR and RES protected these nutraceuticals from degradation, simultaneously preserving their antioxidant activity, and by mixing these NPs with lipid droplets, the bioaccessibility of both encapsulated compounds pronouncedly increased [137]. The incorporation of polysaccharides as a second polymer matrix can provide stability in zein NPs used as DESs for antioxidants in the prevention of chronic degenerative diseases [138].

Ethyl cellulose MPs with encapsulated hydroxytyrosol, a constituent of olive oil showing antioxidant properties, produced by double emulsion solvent evaporation (average particle size ranging from 156.6 ± 6.9 µm to 304.0 ± 16.0 µm) demonstrated the effectiveness of their gastro-resistance and the antioxidant capacity preservation of >50%, indicating possible applications of this formulation in foods, drugs, and nutraceuticals [139]. *Citrus reticulata* Blanco cv. unshiu peel extract (CPE) flavonoids encapsulated by pectin NPs with particle size 271.5 ± 5.3 nm released only 28.78% of flavonoids in simulated gastric fluid within 2 h compared to naked CPE and showed higher antioxidant activity than blank pectin NPs and free CPE [140]. The replacement of 30% of pectin (low charge density) with alginate (high charge density) forming shell around zein NPs significantly improved the aggregation stability at pH 5–7 and high ionic strengths (2.0 µM NaCl), and CUR encapsulated in these core-shell NPs was characterized by higher antioxidant and radical scavenging activities than CUR dissolved in ethanol solutions [141].

The comparison of liposomes and CS coated liposomes co-loaded with vitamin C and FA with the mean particle size of 138 nm and 249 nm, respectively, showed the higher EEs of both drugs as well as the higher antioxidant activity of CS coated liposome nanoformulation, suggesting that it could be applied as a promising DES in the food industry [142]. The deposition of CS and alginate layers on CUR NEs improved CUR antioxidant capacity during in vitro digestion and showed a better control of the rate and extent of lipid digestibility by decreasing free fatty acids release compared to uncoated NEs [143].

### 3.2. Supplements with Antioxidant Effect

**Figure 2:**
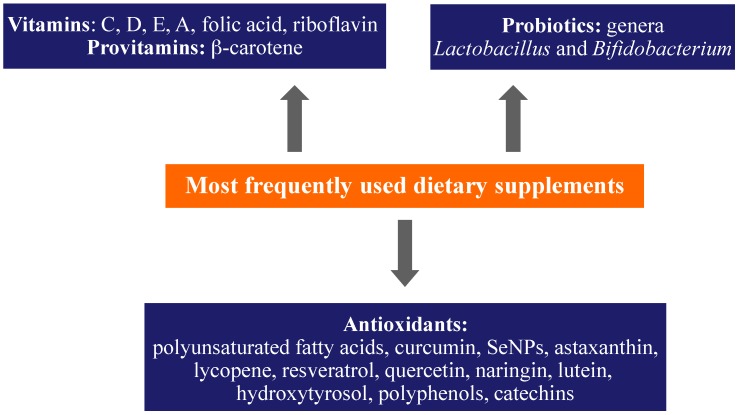
Most frequently used human dietary nanosupplements.

Oxidative stress is able to generate an imbalance between the production and accumulation of ROS in cells and tissues and thus modify the ability of a biological system to detoxify these reactive products. ROS have several physiological roles (i.e., cell signaling), and they are normally generated as by-products of oxygen metabolism; despite this, environmental stressors (i.e., UV, ionizing radiations, pollutants, and heavy metals) and xenobiotics (i.e., antiblastic drugs) contribute significantly to ROS production, thus causing an imbalance that leads to cell and tissue damage (oxidative stress) [144,145]. At present, various oral (flavonoids, carotenoids, vitamin C, and synthetic) antioxidants, see Figure 2, are available on the market and are generally recommended to be used. However, these supplements should be used in accordance with recommendations of a conscious physician or health care professional to avoid their adverse effect—pro-oxidant activity depending on the specific set of conditions (their dosage, redox conditions, the presence of free transition metals in cellular milieu) [144,145,146,147,148]

An improvement in aqueous solubility, antioxidant and other health-promoting properties, in vitro GI release profile, and protection against process and environment harsh conditions (e.g., light, oxygen, high temperatures, and humidity) of hydrophobic food bioactive compounds suitable as DISs could be achieved by nanoencapsulation using different nanoencapsulation DESs, including inclusion complexes of CDs, amylose, yeast cells, nanogels, NEs, nanofibers, nanosponges, nanoliposomes, and NPs made with lipids [149].

A CUR-β-CD inclusion complex and iron oxide NPs were co-encapsulated in liposomes, and these CUR-in-β-CD-in-nanomagnetoliposomes with mean particle size 67 nm and 71% CUR EE showed a radical scavenging property exceeding that of conventional CUR liposome and iron oxide NPs [150].

β-Car, the most important dietary source of provitamin A, is necessary for optimum human health. To increase its solubility and bioaccessibility, 0.1% β-Car was dispersed in corn oil (5 or 10%) and homogenized with 2% SCas solution at 100 MPa, and the prepared NEs had particle sizes <200 nm. β-Car stability towards oxidation decreased with the decreasing droplet diameter, and the extent of lipolysis in an in vitro system was higher and linearly related to the inverse of the droplet diameter [151]. The updated understanding of emulsion-based DESs for β-Car was overviewed by Mao et al. [152] who focused their attention also on emulsion design enabling the delivery of β-Car in complex food systems and fulfilling its benefits in functional foods. Lipid droplets in β-Car enriched O/W emulsions stabilized with surface-active chlorogenic acid-lactoferrin-polydextrose conjugate used as an emulsifier with the mean particle diameter of <400 nm across the pH range 2–9 (except pH value around 6.0) exhibited better stability against droplet aggregation under simulated GI tract conditions (mouth, stomach, and small intestine) than other systems, which resulted in improved β-Car bioaccessibility, and such formulations could be potentially applied as protectors and carriers of hydrophobic drugs, supplements, and nutraceuticals [79]. Encapsulation of β-Car into solid lipid MPs of palm stearin stabilized with hydrolyzed soy protein isolate and containing α-tocopherol with the mean diameter of about 1.2 μm preserved approx. 75% of the encapsulated β-Car after 45 d of storage, and the formulation withstand treatments with higher temperatures (>60 °C), while showing low stability after different ionic strength stresses [153].

DESs of Q, one of the most well-known flavonoids that was included in human diet long ago due to health benefits associated with its antioxidant, anti-inflammatory, antiviral, and anticancer activities as well as Q biological activities themselves, its chemical stability, metabolism, and positive impact on some cardiovascular diseases (CVD) (i.e., heart disease, hypertension, and high blood cholesterol) were overviewed by Wang et al. [154]. An extract of tartary buckwheat rich in flavonoids (TBFs) incorporated in spherical biocompatible lipid–polymer hybrid NPs of 61.25 ± 1.83 nm showed higher antioxidant activity and significant suppression of the pro-inflammatory cytokine secretion in RAW 264.7 macrophage compared to free TBFs and exhibited immune-enhancing efficacy in immunosuppressed mice, suggesting that such nanosystem loaded with TBFs is suitable for nutraceutical applications [155]. Nanoliposomes incorporating olive leaf extract containing high levels of phenolic compounds and oleuropein, showing antioxidant and antimicrobial activities, with average particle size 25–158 nm, negative charge, and EE 70.7–88.2%, which were supplemented to yogurt, improved its antioxidant activity, and no significant changes in color and sensorial attributes were observed, suggesting that olive leaf phenolics can be entrapped in nanoliposomes and could increase the nutritional value of products like yogurt [156].

RES encapsulated in zein/pectin core-shell NPs with mean diameter approx. 235 nm and polydispersity index 0.24 showed improved in vitro antioxidant activity as well as lower IC_50_ values (by 32%) related to antiproliferative activity tested using human hepatocarcinoma Bel-7402 cells compared to free RES, suggesting that such nanoformulation of RES could be used in functional foods and beverages as well as in DISs and pharmaceutical products [157]. The aqueous solubility of RES from α-lactalbumin (α-Lalb)-RES nanocomplexes was 32-fold higher than that of free RES, and the nanocomplexes considerably improved the antioxidant chemical stability under storage, especially at pH 8.0 and high temperature, and showed superb in vitro antioxidant activity compared to free RES, suggesting that α-Lalb as a nanoscale carrier could effectively deliver lipophilic nutraceuticals in the functional food, biomedical, and pharmaceutical products [158]. The protection of light-sensitive AST, a carotenoid with the most potent antioxidant activity, from photodegradation achieved by its inclusion in different hierarchically assembled nano- and microstructures in order to produce model foods for humans and fishes decreased as follows: NEs > carrageenan-coated NEs > CS coated NEs, and CS beads provided higher protection to AST than alginate beads. These hierarchically assembled materials represent ideal platforms to create foods for humans and animal species, because their flexibility enables also the incorporation of other active molecules such as proteins, PUFAs, antibiotics, antiparasitics, etc. [159]. The average size (94 nm) of AST-loaded NLCs containing α-tocopherol and ethylenediaminetetraacetic acid as antioxidants and stabilized with Tween 80 and lecithin increased at pH ≤ 5, high NaCl concentrations (≥50 mM), and slightly at simulated gastric juice, which was connected mainly with decreasing zeta-potential, while increasing at treatment at 80/90 °C. On the other hand, cryoprotectant glycerol prevented the aggregation of AST-NLCs during freeze-thawing. Therefore, this nanoformulation could be used as a DIS [160].

Nanoscale thymoquinone [161], which was found to improve the anticancer roles of doxorubicin by upregulation of P53 and downregulation of Bcl2 and potentiate paclitaxel’s apoptosis in MCF-7 breast cancer cells, could protect also against diabetes, inflammation, central nervous system, and hepatotoxicity primarily by enhancing the antioxidant status of organs and could be considered as a promising nutraceutical for human health [162].

It should be noted that microencapsulation of riboflavin, a water-soluble vitamin acting as cofactor in various processes of oxidation-reduction in a cellular system, with galactomannan biopolymer and Pluronic^®^ F127 resulted in its slower release in both acidic and basic media compared to free vitamin [163].

Digested kenaf (*Hibiscus cannabinus* L.) seed O/W NEs stabilized by a SCas, Tween 20, and β-CD complex demonstrated good lipid digestion, significant bioaccessibility of antioxidants (tocopherols and total phenolic contents), and lower phytosterol degradation rate compared to digested bulk oil, which indicates the possibility of their future application in food and nutraceutical industries [164].

## 4. Other Functional Applications of Human Supplements

This chapter is focused on all other applications of DISs and FSMPs except antioxidants, i.e., supplements affecting intestine, various nutraceuticals with beneficial effect against tumor cells or constituting nutritional support therapy in the treatment of cancer, supplements supporting mental and psychomotor development, supplements for prophylaxis of metabolic syndrome, supplements for osteoporosis management, and supplements against iron deficiency.

### 4.1. Supplements Affecting Intestine and Absorption

It is indisputable that for proper functioning of the intestines, nutrient absorption and prevention of malnutrition, it is necessary to have a suitable composition of the “good” intestinal microflora. Different diseases and subsequent treatments may change the composition of the intestinal microflora, which may result in various, initially intestinal problems. These problems can be avoided by using different products. Prebiotics are compounds in food that induce the growth or the activity of beneficial microorganisms. Probiotics are live microorganisms (in general, bacteria of the genera Lactobacillus and Bifidobacterium) intended to provide health benefits when consumed, generally by improving or restoring the gut flora. Synbiotics refer to food ingredients or DISs combining probiotics and prebiotics in the form of synergism [165,166].

Probiotics, prebiotics, and synbiotics could suppress enteric pathogens, because they can compete with pathogenic microbiota for adhesion sites, inhibit the growth of pathogens, or stimulate, modulate, and regulate the immune response of the host by initiating the activation of specific genes in and outside the intestinal tract. Moreover, it was also shown that probiotics regulate fat storage and stimulate intestinal angiogenesis [167]. Probiotic encapsulation technology was developed rapidly in the past decade. Based on this technology, a wide range of microorganisms have been immobilized within semipermeable and biocompatible materials that modulate the delivery of cells [168].

Co-encapsulation of probiotic strains *Staphylococcus succinus* (MAbB4) and *Enterococcus fecium* (FIdM3) in alginate (2 g/100 mL) resulted in a significant improvement (*P* < 0.05) in the survival of co-encapsulated cells when exposed to acidic (pH 2.0–3.0) and bile (0.3, 0.6 and 0.8 g/100 mL) conditions. Viability was maintained throughout the storage period and ranged from 8.1 log cfu/mL (Colony Forming Unit) to 7.9 log cfu/mL for about a period of 30 days at 4 °C [169].

Dietary factors such as prebiotics (e.g., inulin) play important roles in the growth of intestinal microbiota and may impact the intestinal health [170]. The encapsulation of the probiotic *Pediococcus pentosaceus* Li05 in alginate-gelatin microgels loaded with MgO NPs enhanced its viability by filling pores inside the microgels and thus, the ability of O_2_ and H^+^ ions to access the probiotic could be inhibited due to the neutralization of H^+^ ions in the gastric fluids by MgO NPs, thereby suppressing its acid-induced degradation. Such formulation could be considered as an appropriate DES for improving the efficacy of orally administered probiotics [171]. The effect of a prebiotic matrix consisting of inulin in concentrations 0%, 5%, 10%, 15% and 20% (*w*/*v*) in alginate beads on the viability of encapsulated probiotic strains *Pediocucus acidilactici*, *Lactobacillus reuteri*, and *Lactobacillus salivarius* was investigated by Atia et al., and the researchers found that the beads with 5% *w*/*v* inulin were the most effective in bacterial protection against bile-salts and acidity [172].

Probiotics are also affected by prebiotics apart from other things. Therefore, Peredo et al. [173] investigated the influence of natural prebiotics potato starch, *Plantago psyllium*, and inulin co-encapsulated with alginate on the viability of *Lactobacillus casei* Shirota and two strains of *Lactobacillus plantarum* Lp33 and Lp17. The results showed a higher encapsulation yield when *P. psyllium* (94% for Lp17) and inulin (78% in Lp33) were used; *P. psyllium* ensured a higher viability of the bacteria during storage at 4 °C and the best protection in GI conditions.

Recent findings related to the production of probiotics, prebiotics, and nutraceuticals using a nanotechnology approach with respect to the functional foods was presented by Mishra et al. [174].

### 4.2. Anticancer Nutraceuticals

Nutraceuticals, such as soya bean, garlic, ginger, green tea, propolis, honey, RES, Q, EGCG, etc., may have chemopreventive effects. They are able to induce the apoptosis of cancer cells. These special foods can be used for chemoprevention or as a supportive therapy at treatment of tumor by standard anticancer chemotherapeutics [175,176,177,178,179,180].

In a review paper, McClements and Xiao [181] focused their attention on some most important anticancer nutraceuticals found in foods, the main factors affecting their bioaccessibility, absorption, and transformation, and different types of DESs and excipient systems improving the overall bioavailability of anticancer nutraceuticals.

(-)-Epigallocatechin-3-gallate is the most abundant catechin and also the most effective cancer chemopreventive polyphenol in green tea. This EGCG pronouncedly inhibited β-Car degradation in both MCT and corn O/W emulsions in a dose dependent manner and did not adversely affect lipid oxidation, while α-Lalb was not able to protect β-Car in MCT emulsions; their combination had a similar effect as EGCG alone [182]. A comprehensive review related to findings concerning the encapsulation of EGCG by means of nanocarriers was presented by Granja et al. [183].

Biopolymer core-shell NPs consisting of hydrophobic protein (zein) as the core and a hydrophilic polysaccharide (pectin) as the shell fortified with CUR showing strong anticancer activity with the diameter of 250 nm, which were converted into a powdered form resulting in good water dispersibility, were reported to be suitable for incorporating CUR into functional foods and beverages as DISs, and pharmaceuticals [184]. Bioavailable NEs loaded with nutraceuticals (CUR and fresh and dry tomato extracts rich in lycopene) with the hydrodynamic size of NEs approx. 100 nm applied in combination with doxorubicin enhanced cell viability in cardiomyoblasts (H9C2 cells) by 35–40% compared to that observed in cardiomyoblasts treated with doxorubicin alone, provided protection against oxidative stress, inhibited the release of IL-6, IL-8, IL-1, TNF-α, and nitric oxide by approx. 35–40%, and increased IL-10 production by 25–27% compared to cells without NE treatment. The best cardioprotective profile was showed by a lycopene-rich NE capable to effectively protect against doxorubicin-induced cardiotoxicity by reducing inflammation and lipid oxidative stress [185].

Both a cinnamon oil NE and a vitamin D encapsulated cinnamon oil NE with particle sizes 40.52 and 48.96 nm, respectively, arrested the cell cycle progression in the G_0_/G_1_ phase, showed an increased expression of Bax, capase3, and caspase-9, and decreased the expression of BcL2 proteins along with a considerable increase of apoptotic cell population and loss of mitochondrial membrane potential. The NE with cinnamon oil as a carrier for a lipophilic nutraceutical like vitamin D showing potential anticancer activity in human alveolar carcinoma cells could be also used in the food industry [186].

A nanonutraceutical formulation of ω-3 PUFAs (fish oil) could effectively inhibit the release of ROS and reactive nitrogen species from human neutrophils and murine macrophages, the production of the proinflammatory cytokines TNF-α and MCP1, and tumor-cell proliferation in FaDu head and neck squamous carcinoma and 4T1 breast cancer cells in in vitro cultures. The ω-liposomes, in which docosahexaenoic acid (DHA) was formulated, could be used for intravenous delivery of fish oil fatty acids resulting in beneficial effects in the treatment of inflammatory disorders and cancer [187]. It is known that DHA (ω-3 PUFA), a component of fish oil, suppresses rat mammary carcinogenesis, reduces cell growth, and induces apoptosis in human breast cancer cell lines. An acid stable liposome formulation of DHA with the use of ether and phytanyl lipids similar in structure to those found in Archaea having the mean particle size of 137 ± 12 nm and a slightly negative charge was resistant to oxidation and stable over the pH range of 1.0–7.4 at 37 °C for two hours. Cell viability in MCF-7 cells and apoptosis in both MCF-7 and MDA-MB-231 cells were reduced more effectively by this liposomal formulation than by free DHA, suggesting that it could be potentially used in breast cancer prevention [188].

The investigation of nanosized complexes prepared using high amylose corn starch and flax seed oil processed to powder of MPs by spray-drying and subsequently incorporated into bread formulation showed a considerable reduction of lipid oxidation in breads during baking due to the encapsulation as well as a decreased formation of carcinogen acrylamide, suggesting a beneficial effect of this nanoformulation on the final product quality and safety [189].

### 4.3. Supplements Supporting Mental and Psychomotor Development

Fermented soybean nanonutraceuticals administered to rats intoxicated with colchicine and showing impairment in learning and memory and decreased activity of acetylcholinesterase (AChE) caused an increase of AChE activity (42%), a reduced activity of GSH (42%), SOD (43%), and catalase (41%), and decreased lipid peroxidation (28%) and protein carbonyl contents (30%), which suggests a possible neuroprotective efficiency of the nanonutraceuticals, and in addition, a significant amyloid-β and BACE-1 inhibition activity was demonstrated in an in silico study. The beneficial effect of the discussed nanonutraceuticals is associated with their strong antioxidant activity, and it could be assumed that they could also positively influence cognitive defects associated with Alzheimer’s disease [190].

Encapsulation in bovine-milk exosomes could protect cargos against enzymatic and nonenzymatic degradation. RNAs encapsulated in exosomes could be delivered to circulating immune cells in humans, and some microRNAs and mRNAs in bovine-milk exosomes could regulate human gene expression and be translated into protein. Gene expression can be altered by low concentrations of dietary microRNAs through noncanonical pathways, such as the accumulation of exosomes in the immune cell microenvironment and microRNA binding to Toll-like receptors. In mice, the proliferation of intestinal cells was promoted by porcine-milk exosomes, suggesting that milk exosomes and their cargos could be used in human nutrition. Therefore, it was suggested that milk modified in this way could contribute to better mental, psychomotor, and functional development of infants [191].

Natural compounds that are commonly present in foods and beverages are regarded as promising molecules in a nutraceutical approach associated with life-long healthy diets. An increased attention is devoted to food molecules that are candidates to enter clinical trials as such or after targeted molecular engineering and could have a beneficial effect on amyloid neurodegenerative diseases. Natural phenols abundant in healthy food products, such as green tea, red berries, extra virgin olive oil, red wine, and spices, could be considered particularly promising [192]. Biodegradable poly(lactic-co-glycolic acid) NPs encapsulating ginsenoside Rg3 (an important constituent of ginseng, playing a significant role in memory and improving cognition) and thioflavin T, which showed neuroprotective effects, were reported to be a theranostic material for the detection and treatment of Alzheimer’s disease [193].

### 4.4. Supplements for Metabolic Syndrome Prophylaxis

Sodium alginates could be used for the management of GI tract disorders and the attenuation of components of the metabolic syndrome such as obesity, type 2 diabetes, hypertension, non-alcoholic fatty liver disease, and dyslipidemia. They could also protect cells during transplantation from immune responses of the host, and, in combination with antacid alginates, be applied in the treatment of gastric reflux disease. Moreover, alginates decrease food intake by inducing satiety, increase weight loss in patients on a calorie-restricted diet, and reduce both glucose and fatty acid uptake, and a decrease in blood pressure by alginates in rat models of hypertension was reported as well [194].

Using advanced proteomic and bioinformatic approaches, Kar et al. [195] characterized the protein components of six different protein sources (casein, partially delactosed whey powder, spray-dried porcine plasma, soybean meal, wheat gluten meal, and yellow meal worm) and predicted the bioactive properties of these protein sources after in silico digestion with monogastric proteolytic enzymes. The tested protein sources were potentially rich in bioactive peptides, in particular, angiotensin-converting enzyme inhibitors and peptides with antioxidant properties, and could be used as alternative sources of protein in animal feeds for monogastrics.

Temporal improvements in vitamin D status by vitamin D supplementation resulted in an increase in serum 25-hydroxyvitamin D concentrations and reduction of serum homocysteine concentrations suggesting that such treatment could reduce risk factors for CVD and may potentially contribute to the primary prevention of CVD [196]. Liposome-in-alginate beads were used to encapsulate the oyster hydrolysates showing antihypertensive effect to improve their bioavailability, protect them from degradation, and obtain sustained release; the release time of the oyster hydrolysate in the simulated GI fluid was up to 16 h [197]. Encapsulation of naringin, a flavonoid that occurs naturally in citrus fruits and possesses strong health benefits (recommended for the prevention of CVD and diabetes), in ternary NPs consisting of amylose, α-LLA, and β-Lglb resulted in a gradual release of naringin from the ternary NP–naringin inclusion complex in simulated gastric and intestinal fluids, and ternary NPs effectively improved the bioavailability of bioflavonoid [198].

Mahmoud et al. [199] studied the impact of dietary camel whey protein administered as a supplement to streptozotocin (STZ)-induced diabetic pregnant mice on the efficiency of the immune system of the offspring and verified its protective role in decreasing the tendency of the offspring to develop diabetes and related complications. A comparison of prophylactic effects of α-eleostearic acid rich nano and conventional bitter gourd seed oil emulsions in induced diabetic rats showed that the maximum efficiency in suppressing oxidative stress was achieved with a diet supplementation of 0.5% (*w*/*v*) NE with bioactive lipid-conjugated α-LNA, suggesting that such nanoformulation could be used as an appropriate nutraceutical against diabetes mellitus strongly attenuating an adverse impact of excessive ROS [200].

Although natural nanosized clinoptilolite and/or metformin did not affect pronouncedly the levels of serum glucose, minerals, and lipid profile in rats with high-fat-diet/STZ induced diabetes, the co-treatment of clinoptilolite with the drug notably increased high-density lipoprotein (HDL) cholesterol, while Cu and Ca levels increased only in the metformin group [201]. In STZ induced diabetic rats treated with nanosized clinoptilolite, blood glucose was found to decrease to near normal levels (12.4 vs. 27.5 mmol/L), but no significant impact on oxidative stress markers was estimated [202]. Nanosized clinoptilolite injected to STZ induced diabetic rats caused a partial improvement in their weight status and lack of undesirable effects, although beneficial changes in lipid profile were not detected, which could be connected with short study duration [203].

CS NPs loaded with *Stevia rebaudiana* leaf extract caused a considerable decrease of the mean fasting blood glucose level of treated diabetic rats in comparison with the diabetic control group, and serum levels of different enzymes and some antioxidants, e.g., catalase, reduced glutathione (GSH), and superoxide dismutase (SOD), were closer to normal levels in the group treated with NPs than in the control group [204].

By adding *Catathelasma ventricosum* polysaccharides (CVPs) to the redox system of selenite and ascorbic acid, spherical CVPs–selenite NPs with particle size approx. 50 nm were prepared, and based on serum profiles and antioxidant enzyme levels, it could be concluded that CVPs–selenite NPs showed a notably higher antidiabetic activity (*p* < 0.05) than other SeNPs, selenocysteine, and Na_2_SO_3_ [205].

To control the release of the anti-hyperglycemic agent fisetin for nutraceutical and/or therapeutic applications, an oral controlled release system consisting of polymeric NPs (140–200 nm) based on poly-(ε-caprolactone) and poly(lactic-*co*-glycolic acid)-polyethylene glycol-COOH encapsulating fisetin was designed, which protected and preserved the release of the active compound in gastric simulated conditions, controlled the release in the intestinal medium, and showed an improved α-glucosidase inhibiting activity of fisetin compared to that of the commercial formulation acarbose [206]. 

NLCs loaded with betasitosterol, a phytosterol showing beneficial effects on reducing total cholesterol and low-density lipoprotein (LDL), with particle size 165 nm, zeta potential −13.5 mV, and EE 99.96%, which were incorporated in butter, showed good stability during three months’ storage period and increased the antioxidant property of enriched butter during the storage period, suggesting the suitability of such nanoformulation for functional dairy products [207]. Multilayer CS–alginate–CUR NEs could be important for functional food development for combating obesity, because they increase satiety by retarding lipid digestion [143].

### 4.5. Supplements for Osteoporosis Management

Many supplements in pharmacies serve to prevent or mitigate the effects of osteoporosis. Calcium (Ca) has clearly been shown to have some positive effect on osteoporosis, although the bioavailability of Ca from classical preparations is approximately 10–15% [208,209,210]. Recently, a number of scientific teams investigated Ca supplementation by nano-Ca either as solid peroral DISs or as nano-Ca from the fortified milk. Experiments performed in vivo on ovariectomized (OVX) rats demonstrated much greater absorption (up to 89%) and overall bioavailability (up to 42%) of preparations with nano-CaCO_3_, citrate, or organically bound in shell oyster. Therefore, by in vivo studies it was confirmed that the application of nanosized Ca could improve Ca and even phosphorous content in bones [211,212,213,214,215].

Ca alginate NPs (200–500 nm in diameter) loaded with collagen peptide chelated Ca with the average diameter of approximately 150 nm and the Ca content of up to 130.4 g/kg notably enhanced Ca absorption and significantly increased femur bone mineral density and femur Ca content in rats, suggesting that they could prevent Ca deficiency and could be used as a new Ca supplement in the food industry [216]. A nanocomposite of whey protein hydrolysate chelated with Ca showed superb stability and absorbability under both acidic and basic conditions, which was beneficial for Ca absorption in the GI tract of the human body. Its pronouncedly higher Ca absorption on Caco-2 cells compared with Ca gluconate and CaCl_2_ in vitro suggested a possible increase in Ca bioavailability and thus its potential to be used as DIS for improving bone health of humans [217].

It is also important to remember that oral administration of Ca hydroxyapatite microcrystals can accelerate fracture healing and repair and even prevent osteoporosis [218]. Moreover, Zhang et al. [219] described the benefit of nanohydroxyapatite/CS composite for bone regeneration when it was administered by injection. In addition, these nanocomposites showed antistaphylococcal activity [220].

Additionally, CaCO_3_ from eggshell can be used as a Ca supplement [221]. Chicken eggshell powder became an attractive source of Ca for human nutrition. It can be added to food or drinks. For example, chocolate cakes were fortified by 3%, 6%, and 9% of them, and the results indicated that with respect to the Ca content, texture and sensory properties of the cakes, 6% eggshell supplementation (i.e., increased Ca content to 816.8 mg/100 g) was the best [222]. The preventive effects of nanopowdered eggshell (NPES) on postmenopausal osteoporosis in OVX rats was also studied, and the results were surprising. NPES fed rats showed an increase in bone mineral densities (BMD) by about 7% compared to OVX rats. Only powdered eggshell led to an increase of BMD by 2%. Serum analysis showed that NPES fed rats had a 22.4% higher osteocalcin level than OVX rats. Therefore, NPES attenuated the bone loss induced by ovariectomy in rats [223]. High-calcium yogurt as food for combat with osteoporosis was prepared using its fortification with 10-nm crystals of NPES. The addition of NPES up to 0.3% gave cow and buffalo’s milk yogurts with acceptable composition, textural properties and sensory attributes, and this additive increased the Ca content of yogurt by about 15% [224].

### 4.6. Supplements against Iron Deficiency

Iron-deficiency anemia is the most common nutritional disorder worldwide with impact on health and economy. In spite of a number of commercially available supplements, this deficiency is a global public health problem due to the poor tolerability of the standard care soluble iron salts (such as ferrous sulfate), which results in non-compliance and ineffective correction of iron-deficiency anemia. On the other hand, poorly water-soluble compounds cause less sensory changes, but are not well absorbed [225]. Nanoformulations of iron were proposed to fortify food and feed to address these issues due to enhanced bioavailability, good product stability, limited side effects and the absence of changes of taste and color of the fortified foods [226]. In addition, in vitro and in vivo experiments have shown that iron NPs can be considered safe [227]. Ferritin, which is well absorbed [228] is itself composed of an iron oxide nanocore surrounded by a protein shell. Recently, Powell et al. [229] synthesized tartrate-modified, nano-disperse ferrihydrite having small primary particle size and enlarged or strained lattice structure (about 2.7 Å for the main Bragg peak versus 2.6 Å for synthetic ferrihydrite) that was able to efficiently provide GI delivery of soluble Fe(III) without the risk of free radical generation in murine models, where GI delivery did not depend on luminal Fe(III) reduction to Fe(II), and absorption was similar to that of FeSO_4_. This nanoformulation could be considered as a potentially side effect-free form of Fe supplementation to human suffering from anemia.

The most promising preparation (iron hydroxide adipate tartrate: IHAT) showed ~80% relative bioavailability to FeSO_4_ in humans and, in a rodent model, IHAT was equivalent to FeSO_4_ at repleting hemoglobin. Moreover, IHAT did not accumulate in the intestinal mucosa and, unlike FeSO_4_, promoted a beneficial microbiota. In an in vitro study, IHAT was 14-fold less toxic than Fe(II) sulfate/ascorbate. The results of IHAT NPs observed from three-arm, double-blind, randomized, placebo-controlled trial conducted in Gambian children 6–35 months of age in relation to ferrous sulfate and non-inferiority in relation to placebo in terms of diarrhea incidence and prevalence confirmed the hypothesis that supplementation with IHAT eliminates iron deficiency and improves hemoglobin levels without inducing GI adverse effects [230,231].

Poorly water-soluble nanosized FePO_4_ with specific surface area approx. 190 m^2^/g made by scalable flame aerosol technology possesses in vivo iron bioavailability in rats comparable to FeSO_4_ and causes less color change in reactive food matrices than conventional iron fortificants. The addition of Zn or Mg oxides to nano FePO_4_ increases Fe absorption and also improves their color [232]. Additionally, Srinivasu et al. prepared nano ferric pyrophosphate (particle size 10–30 nm) as a potential food fortificant in iron-deficiency anemia and found that the peroral bioavailability of ferric pyrophosphate NPs in rats, calculated using hemoglobin regeneration efficiency, was 103.02% with respect to the reference salt, ferrous sulfate, while the NPs did not show any significant toxicity [233].

Salaheldin and Regheb biosynthesized biocompatible Fe_3_O_4_ NPs capped with vitamin C, and thus intestinal villi absorbed the NPs as vitamin C and not as an iron, because iron was coated with vitamin C. Clinical and histopathological studies on rats recommended the use of fortified biscuits with concentrations of 10 ppm and 30 ppm of nano iron; hemoglobin concentration increased from 9.9 ± 1.2 g/dL to 14.6 ± 1.1 and 16.7 ± 1.6 g/dL, respectively [234].

## 5. Veterinary Nanoscale Nutraceuticals and Dietary Supplements

As mentioned above for humans, nanoformulated DISs can also be applied for animals. In general, these veterinary DISs are regulated by the FDA’s Center for Veterinary Medicine [235,236]. Nutraceuticals have become popular with the veterinary community; worldwide estimates of sales approach $100 billion [237]. Therefore, many different products can be found for veterinary applications. For example, the use of clinoptilolite (natural zeolite comprising a microporous arrangement of silica and alumina tetrahedra) showing unique antibacterial properties as a DIS in food and unifying properties of an immunomodulator and nutraceutical could represent an alternative to antibiotic growth promoters in animals of veterinary importance. Valpotic et al. [238] focused their attention mainly on clinoptilolite potentials and limitations in cattle related to metabolic and endocrine status, oxidative stress, and systemic local inflammatory responses involved in reproductive and metabolic disorders of dairy cows.

Zinc (Zn), copper (Cu) and selenium (Se) are essential nutrients for animals and humans, because these metals occur in various metaloenzymes as co-factors [239]. Zn is a nutritionally indispensable trace element that is required for normal growth, bone development, feathering, appetite regulation, metabolic functioning of nearly 300 biochemical enzymes, hormone production, cell division, protein and DNA synthesis for all avian species [239,240]; so, it can affect animals production and reproduction performance [241]. Zinc deficiency in animals caused a decrease in feed intake, growth, serum insulin-like growth factor-I, and growth hormone (GH) and lowered the hepatic production of insulin-like growth factor-I, GH receptor, and GH binding protein [241,242,243,244]. In addition, Zn is used to decrease fermentation of digestible nutrients in intestines and improve nutrients digestibility and appetite. Dietary Zn supplementation stimulates feed intake probably caused by increased ghrelin secretion [245]. It was observed that it caused an increase in insulin-like growth factor expression in the small intestine mucosa [246]. Increased Zn concentration in the intestines influences their structure and function. The growth-stimulating properties of dietary Cu have been attributed to its antimicrobial action, however, it was shown that also intravenous injection of Cu to weanling piglets stimulated their growth [247]. It seems that the growth-promoting properties of high dietary concentrations of Cu complement its antimicrobial action [248].

Se is very important in animal nutrition, because it functions as an anti-oxidant assisted by vitamin E; e.g., Se is a cofactor of glutathione peroxidase (GSHpx), deiodinases, thioredoxin reductases, selenophosphate synthatase, selenoprotein P, selenoprotein W, etc. Se deficiency can be a major problem that can be reduced or prevented by supplementation with inorganic or organic sources of Se. On the other hand, Se in high concentration is toxic to human and animal [249]. Recent knowledge related to beneficial biological effects of SeNPs in the organism, absorption mechanisms, and nanotechnological applications for peroral administration were summarized by Hosnedlova et al. [250]. The applications of the above-mentioned nutrients (Zn, Cu, Se) in nanoscale formulations allow increased efficacy, enhanced absorption, lower overall doses, etc. [251,252]. In the following subchapters, an attention is mainly focused on the beneficial effect of some inorganic NPs (Zn, ZnO, Cu, CuO, Se, Ag) and nanoscale formulations containing organic active compounds (e.g., essential oils, vitamins) on growth performance and some important biochemical parameters of aquatic animals, poultry, pigs and other domestic animals like cattle, sheep, and rabbits. Nanoformulations have also found their way into the fortification of animal feeds [253,254].

### 5.1. Aquatic Animals

The major challenge facing fish farming is the availability of relatively cheap but high-quality feed. Regular fish diet blended with nanosized mineral nutrients has beneficial impact on growth and overall health of fish, because they can pass across the gut tissue into cells more readily than bulk nutrients, and thus their assimilation processes in the fish are accelerated, resulting in improved growth [255].

Thyme essential oil at doses 400 and 800 mg/kg used as a DIS was found to reduce oxidative stress of gibel carp (*Carassius auratus gibelio*; average weight of 8.73 ± 2.1 g), and exposure to a sub-acute toxicity level of AgNPs for a period of 96 h after six weeks of a feeding trial confirmed the resistance of the carp to non-fatal effects of AgNPs [256]. A fish diet containing 1% *Aloe vera* NPs improved the growth factors (weight gain, initial body weight, condition factor, feed conversion ratio, specific growth rate) of Siberian sturgeon [257]. CS NPs showing spherical shape, particle size 185 nm, and positive zeta potential used to carry vitamin C through the GI tract of rainbow trout (*Oncorhynchus mykiss*) exhibited in vivo controlled release until 48 h and increased lysozyme and complement contents in the fish serum [258].

Shrimps (*Litopenaeus vannamei*) reared in clear water and in a biofloc system and receiving feed supplemented with nanocapsules containing lipoic acid showed increased final weight, higher GSH levels in the hepatopancreas and decreased percentage of hyaline hemocytes, while increased levels of granular hemocytes. Increased glutathione *S*-transferase activity in the gills and hepatopancreas was estimated only in shrimps reared in the biofloc system and fed with encapsulated antioxidant, while decreased levels of thiobarbituric acid reactive substances were estimated in the gills and muscles of the shrimps maintained in clear water [259].

A 60-day feeding of red sea bream (*Pagrus major*) with CuNPs (2 mg/kg) or/and vitamin C (800–1200 mg/kg) improved its growth and health, and higher final weight, weight gain, specific growth rate, protein gain, protein retention, feed intake, protease and bactericidal activities, and higher tolerance against stress than in controls was estimated as well. The feed and protein efficiency ratios and the body lipid content were considerably higher at treatment with 0/1200, 2/800, 2/1000 and 2/1200 mg CuNPs/vitamin C per kg, while the application of 2/800, 2/1000 and 2/1200 mg CuNPs/vitamin C per kg resulted in considerably enhanced body protein and higher tolerance against stress compared to the control was estimated as well [260]. Wang et al. [261] reported that for the dietary Cu requirements of Russian sturgeon (9.82 ± 0.08 g) fed with diets containing different forms of Cu for 8 weeks, Cu-methionine (Met) and CuO NPs were 1.5–2-fold more bioavailable than CuSO_4_, optimal doses being approx. 5 mg/kg for Cu-Met or CuO NPs and 8 mg/kg for CuSO_4_.

In *Pangasius hypophthalmus* fed with a diet incorporating 10 and 20 mg/kg ZnNPs and exposed to abiotic stress (sublethal dose of Pb 4ppm and temperature 34 °C), a considerably enhanced growth performance and improved immunological parameters (total protein, Alb, Glb, and Alb/Glb ratio) were observed, and reduced oxidative stress reflected in lower levels of blood glucose, cortisol, and HSP 70 suggested that the supplementation of dietary ZnNPs could alleviate abiotic stress in *P. hypophthalmus* [262]. In Mozambique tilapia (*Oreochromis mossambicus*) receiving a diet supplemented with 0.004% of *Portunus pelagicus* β-1,3-glucan binding protein based ZnO NPs, considerable increases in growth performance and in cellular and humoral immune responses were estimated. Moreover, when after 30 days of a feeding trial, the fish was challenged with aquatic fish pathogen *Aeromonas hydrophila* (1 × 10^7^ cells/mL) through intraperitoneal injection, a reduced mortality rate was observed in fish fed with the diet containing such ZnO NPs, suggesting a potential beneficial impact of the NPs on the immune system and survival of *O. mossambicus* [263]. Beneficial effects of Zn-proteinate, ZnSO_4_, and ZnO NPs applied at dose 50 mg/g of Zn sources in an early diet of rainbow trout larvae with average weight of 82.3 ± 11.6 mg for 70 days enhanced the growth performance of the larvae [264].

Common carp (*Cyprinus carpio*) juveniles (9.7 ± 0.1 g), the diet of which was supplemented with SeNPs (0.7 mg Se/kg), showed the highest weight gain of 97.2 ± 10.8% and feed efficiency ratio 42.4 ± 0.8%, the highest serum hemolytic activity, total immunoglobulin, and total protein and Alb contents as well as the lowest serum total cholesterol and LDL levels after 8 weeks of feeding compared to the carp fed with Na_2_SeO_3_, Se-Met, and the control. Carps fed with SeNPs or Se-Met showed also pronouncedly higher activities of serum glutathione peroxidase (GPx) and SOD and an increase in white blood cell counts, neutrophil percentage, and serum lysozyme activity compared to the control group and the Na_2_SeO_3_ group [265]. Dietary treatments of crucian carp, *Carassius auratus gibelio*, with SeNPs and Se-Met showed higher Se levels in muscle (16.42 ± 1.07 μg/g and 13.52 ± 1.31 μg/g, respectively) compared to carps fed with basal feed (6.10 ± 0.78 μg/g). Although the survival rate and the feed conversion ratio were not affected by the dietary treatments, GPx activities in Se-treated carp plasma and liver differed significantly from those of the control [266]. Dietary SeNPs supplementation at the dose of 0.68 mg/kg to juvenile mahseer (*Tor putitora*) considerably increased red blood cell count, hemoglobin level, hematocrit values, and lysozyme activity as well as serum GH levels, tissue total protein content, and GPx activity in liver and muscle tissues of *T. putitora* [267]. Chinese mitten crabs (*Eriocheir sinensis*) fed with a diet containing 0.2 mg/kg SeNPs in a 60 d feeding trial had a considerably higher weight gain rate and a reduced feed coefficient. When juvenile Chinese mitten crabs were kept under the condition of hypoxia, the up-regulative effects of SeNPs on antioxidant capacity, hemocyte counts, and hemocyanin expression were further amplified. Hypoxia exposure increasing mortality in crabs infected with *A. hydrophila* bacteria was also alleviated when crabs received a diet containing 0.2 mg/kg SeNPs, suggesting the importance of dietary SeNPs in regulating the immunity and disease resistance in crabs kept under hypoxia stress [268]. Naderi et al. [269] who investigated the impact of dietary SeNPs (1 mg/kg), vitamin E (500 mg/kg), and their combination on the humoral immune status and serum parameters of rainbow trout under high-density condition (80 kg/m^3^) reported that the positive effects observed in the performance following the combine treatment may be due to vitamin E alone, because supplementation with SeNPs did not markedly affect the performance in rainbow trout under high-density conditions. In addition, the immuno-protective role of biologically synthesized dietary SeNPs applied at the dose of 1 mg/kg against multiple stressors (Pb level of 4 ppm, high temperature of 34 °C) in *Pangasinodon hypophthalmus* was reported by Kumar et al. [270].

### 5.2. Poultry

Typical poultry diets are commonly enriched by feed additives containing vitamins and minerals to support rapid growth and a favorable feed conversion ratio, and nanosized feed additives characterized by a high surface area to volume ratio and high absorption in the body could be incorporated in vaccines and nutrient supplements and directly transported to targeted organs or systems without degradation resulting in health benefits. Current state of NPs use as poultry feed supplements was reviewed by Gangadoo et al. [271].

AgNPs received in drinking water containing 1000 mg AgNPs/kg significantly reduced the body weights of the broilers after 42 days of administration, and this adverse effect could not be mitigated with a basal diet supplemented with Zn (60 and 120 mg/kg) and vitamin E (α-tocopherol acetate; 100 and 200 mg/kg). On the other hand, the increased activity of CuZn-SOD observed in AgNPs-treated broilers was not recorded in birds fed with the basal diet supplemented with 200 mg/kg vitamin E, suggesting its antioxidant effect. Moreover, Zn supplementing enhanced catalase and GPx activities in the jejunal mucosa resulting in increased malondialdehyde (MDA) levels in the animals. Therefore, it could be concluded that the dietary Zn and vitamin E supplementation was able to attenuate intestinal oxidative stress in AgNPs-treated broiler chickens, although it did not mitigate the growth reduction caused by AgNPs [272]. In ovo feeding was found to reduce post-hatch mortality and skeletal disorders and increase muscle growth and breast meat yield. Sawosz et al. [273] used AgNPs as a protective carrier for adenosine triphosphate (ATP) as well as an active agent, which may penetrate tissues and cells and localize inside cells. They injected AgNPs, ATP, or a complex of AgNPs + ATP (AgNPs/ATP) in broiler eggs, and on day 20 of incubation, the embryos were evaluated. An increased expression of fibroblast growth factor 2, vascular endothelial growth factor, and Na^+^/K^+^ transporting ATPase were estimated at the application of ATP or AgNPs to chicken embryos. Moreover, AgNPs also upregulated the expression of myogenic differentiation 1, affecting cell differentiation. Based on the above-mentioned findings, it could be concluded that an extra energy source in the form of ATP addition enhanced molecular mechanisms of muscle cell proliferation, and ATP and AgNPs could accelerate the growth and maturation of muscle cells [273].

The in ovo injection of CuNPs using the dose of 50 mg/kg CuNPs improved broiler performance more efficiently than the injection of 50 mg/kg CuSO_4_ or the provision of CuNPs or CuSO_4_ in drinking water containing 20 mg/kg CuNPs or CuSO_4_ to growing chickens. In another experiment, which was carried out with 126 one-day-old broiler chickens from day 1 to 35 post-hatching, the in ovo application of Cu enhanced the final body weight, average daily gain, and feed conversion ratio compared to control animals and resulted in a considerable improvement in energy and nitrogen utilization, mainly for CuNPs application. The CuNPs treatment also reduced cholesterol, urea, and glucose levels in the blood [274]. The supplementation of a Cu deficient basal diet of chickens with CuNPs in drinking water to the level of Cu exceeding the National Research Council (NRC) recommendation by 54% resulted in the increased antioxidant potential of the organism and the inhibition of lipid peroxidation. Antioxidant and immune defenses of chickens were simultaneously increased in chickens receiving diet supplemented with CuNPs up to 12 mg per bird during 6 weeks of feeding, i.e., up to a level exceeding the NRC recommendation for growing broiler chickens at the most by 7%. It could be mentioned that at a higher CuNPs supplementation, a deterioration in red blood cell parameters and the stimulation of the immune system reflected in an increase in interleukin-6, immunoglobulin A (IgA), IgM, and IgY was observed [275].

The in ovo administration of NPs could be considered as a new method of nano-nutrition to supply an additional quantity of nutrients to embryos. ZnNPs, CuNPs and SeNPs supplemented in ovo at doses 20, 40, 60, and 80 μg ZnNPs/egg, 4, 8, 12, and 16 μg CuNPs/egg, and 0.075, 0.15, 0.225, and 0.3 μg SeNPs/egg (18th day incubation, amniotic route) did not show any adverse effect on the developing embryo and did not influence the hatchability, best feed efficiency being observed with 40 μg ZnNPs/egg, 4 μg CuNPs/egg and 0.225 μg SeNPs/egg. Moreover, the application of 12 μg CuNPs/egg resulted in considerably higher breast muscle percentage [276].

In white Leghorn laying hens (68-week old) receiving a diet supplemented with Zn-Met, bulk ZnO, and ZnO NPs reaching the level of 60 mg Zn/kg in the diet, pronouncedly higher Zn retention, serum GH concentration, and carbonic anhydrase activity were observed at the application of the ZnO NPs and Zn-Met compared to the control, and the ZnO NPs enhanced eggshell thickness as well [241]. Laying hens at 64 weeks of age fed with a basal diet supplemented with 80 mg/kg of bulk ZnO, ZnO NPs, and Zn-Met showed considerably higher egg production and egg mass as well as SOD activity in the liver, pancreas, and plasma when Zn-Met and the ZnO NPs were applied, while the greatest increase in eggshell thickness and shell strength was observed at the ZnO NPs application. The Zn supplementation resulted in reduced egg loss and lower MDA content and had a beneficial effect on serum total protein, Alb, glucose, alkaline phosphatase activity, carbonic anhydrase activity, and Zn level, which was reflected in an improved performance of laying hens. Due to the enhanced Zn absorption in the intestine of aged layers at the application of ZnO NPs, they could be considered as a more suitable source of Zn in diets than bulk ZnO [277]. At the dietary supplementation of Zn-Met, ZnO, ZnO NPs or polyglutamic acid (PGA)–ZnO NPs reaching the level of 80 mg Zn/kg in the diet, increased Zn content in eggshells, serum Zn concentration, ghrelin and IgG levels of 64-week old brown layers were observed at the application of the ZnO NPs and the PGA-ZnO NPs, exceeding that observed at the application of bulk ZnO, and serum carbonic anhydrase activity and ghrelin levels were also increased compared to Zn-Met, suggesting that the ZnO NPs alone or in combination with PGA show beneficial impact on the Zn status of aged layers [278].

Investigation of the effects of SeNPs on performance, meat quality, immune function, oxidation resistance, and tissue Se content in broilers performed with 1-day old male Arbor Acres broilers showed that the supplementation of corn-soybean meal-based diets with 0.3–0.5 mg SeNPs/kg was found to be the best, and the maximum supplementation of SeNPs could not exceed 1.0 mg SeNPs/kg [279]. The adverse effects of oxidative stress in broiler chickens induced by *tert*-butyl hydroperoxide were attenuated when the animals received a diet supplemented with 0.3 mg SeNPs/kg. In stressed chicks fed with SeNPs, the heterophil:lymphocyte ratio was lower than in the groups, the diet of which was supplemented with bulk inorganic or organic Se, suggesting a higher effectiveness of SeNPs in the mitigation of oxidative stress [280]. Supplementation of SeNPs (0.1–0.5 mg/kg) in broiler diets could improve growth performance, carcass components, and immune function of the animals, and no adverse effects on internal organs, other carcass parameters, and GI parts were observed. SeNPs dietary supplementation resulted in significantly improved weight gain and feed conversion ratio during the whole period of experiment (42 days) and more efficient energy and protein utilization compared to the control group [281].

Rahmatollah et al. [282] reported that 1.2 mg/kg cysteine-coated Fe_3_O_4_ NPs were found to be required and sufficient for quails’ optimal maintenance and growth suggesting that cysteine-Fe_3_O_4_ NPs can be used as a Fe source in the quail diet.

Cr utilization in 32 three-week-old broilers fed with a diet supplemented with Cr at the 1200g/kg level using CrCl_3_, chromium picolinate (CrPic), and CrPic NPs decreased as follows: CrPic NPs > CrPic > CrCl_3_ > control groups, and significant differences between individual groups were estimated. When one-day-old broilers were fed with diet supplemented with the above-mentioned Cr compounds, the feed intake of 4–5 weeks showed better results in the CrCl_3_ group compared to the CrPic group, while the LDL-cholesterol in the CrPic NPs groups was lower than in the CrPic group, and CrPic NPs and CrPic groups showed considerably enhanced serum Cr concentration compared to the control and CrCl_3_ groups. Based on the above results, it could be concluded that the CrPic NPs supplementation has advantages compared to the bulk CrPic supplementation, because it not only increases Cr utilization but also results in a lower serum LDL-cholesterol level in broilers [283].

### 5.3. Pigs

ZnO and Cu salts, traditionally used in high doses as supplements to piglet’s diet, stimulate piglet’s daily gain and decrease feed conversion factor. However, the application of high concentrations of these metal additives could result in increased environmental pollution of soil and tap water; on the other hand, Zn applied at doses 2500–3000 mg/kg feed can contribute to the development of antimicrobial resistance and may regulate the expression of genes that modify piglets’ immune response. Consequently, higher bioavailability which could be achieved by applying nanosized ZnO/Cu particles could notably reduce the dietary inclusion rate and environmental pollution with preserving beneficial impact on pig’s health [244].

The degree of the reduction of piglet diarrhea incidence observed with a low dose of ZnO NPs (600 mg Zn/kg) supplemented to the basal diet of weaning piglets was comparable with that observed with the dose of 2000 mg Zn/kg when bulk ZnO was used, which could be connected with improved intestinal microbiota and inflammation response in piglets at the ZnO NPs application. Moreover, the application of ZnO NPs could contribute to reduced Zn environmental pollution [284]. ZnO NPs used as a DIS increased Zn digestibility, serum GH levels, and carbonic anhydrase activity and enhanced the immune response of weanling piglets [285].

Cr(III) belongs to essential elements in the nutrition of both animals and humans, and in many animal species its deficiency results in reduced feed intake, lower weight gains, reproductive disorders, and increased lipid levels, and a moderate Cr deficiency represents a risk factor of ischemic heart disease with myocardial infarction and coronary artery disease. Cr improves lean body mass in animals, increases growth rate and feed conversion, and improves feed intake and energy efficiency, and the dietary Cr requirement of an animal body is probably 300 μg Cr/kg d.w. of feed. A pronouncedly increased Cr content in the blood, longissimus muscle, heart, liver, kidneys, jejunum, and ileum was observed in pigs receiving dietary Cr nanocomposite supplementation [286]. Dietary CrPic NPs supplementation at 400 ppb increased feed intake in finisher gilts during mid-summer and was able to improve some of the adverse effects of heat stress in pigs, through decreasing circulating cortisol levels [287]. Pigs with initial body weight of 66.10 ± 1.01 kg receiving basal diet supplemented with 200 or 400 μg/kg of Cr from Cr-loaded CS NPs (Cr–CS NPs) for 35 d showed increased carcass lean ratio and longissimus muscle area, decreased carcass fat ratio and backfat thickness as well as increased serum free fatty acids, lipase activity, and serum insulin-like growth factor I, while a decreased level of serum insulin was estimated. Moreover, decreased activities of fatty acid synthase and malate dehydrogenase and increased activity of hormone-sensitive lipase in subcutaneous adipose tissue were observed in treated pigs. These results indicate a favorable impact of Cr in the form of the Cr–CS NPs on growth, carcass characteristics, pork quality, and lipid catabolism in finishing pigs [288]. The control diet supplemented with 200 μg Cr from Cr nanocomposite pronouncedly reduced serum levels of glucose, urea nitrogen, triglyceride, cholesterol, and nonesterified fatty acid, increased total protein, HDL, and lipase activity in finishing pigs with initial weight 64.8 ± 0.83 kg fed for 35 d, and considerably increased serum insulin-like growth factor I, while reducing serum insulin and cortisol levels. Moreover, it affected immune status in finishing pigs, which was reflected in notable increments of IgM and IgG contents in plasma [289].

### 5.4. Other Pets

Dried matrices of CS Cu chelate gels designed as a multimicronutrient feed additive for cattle were loaded with vitamin riboflavin. Following restricted rehydration in simulated rumen fluid, they exhibited sustained release of riboflavin without releasing Cu in these neutral conditions for up to 24 h, demonstrating Cu rumen bypass. A sustained release of the mineral was observed in abomasal conditions of pH 2 over a 3 h period suggesting that this formulation could supply nutritionally relevant levels of the free mineral in these conditions, as required for effective supplementation in cattle [290].

A decreasing trend in serum Fe concentration was observed in Lori-Bakhtiary sheep, which orally received SeNPs and Na_2_SO_3_ (1 mg/kg) for 10 consecutive days, particularly during the early and middle stages of supplementation (0–20 days) in contrast to the increasing levels of total iron binding capacity, suggesting that the expression of transferrin and its receptor genes was considerably increased. However, after this period, the expression of the transferrin and transferrin binding receptor genes showed a notable decrease, especially in SeO_3_^2−^ treated animals [291].

Improved rumen fermentation and feed utilization, stimulation of rumen microbial activity, digestive microorganisms, and enzyme activity by supplementation of SeNPs in basal diet of sheep with optimum dose approx. 3.0 g/kg dietary dry mater was reported by Shi et al. [292]. Using the rumen simulation technique, it was observed that nanoemulsified soyabean oil modulated the PUFAs proportions in ruminal cultures, which was reflected in markedly increased proportions of oleic acid, α-LLA, and α-LNA in the fermentation fluid without any negative effect on rumen fermentation parameters [293].

**Figure 3:**
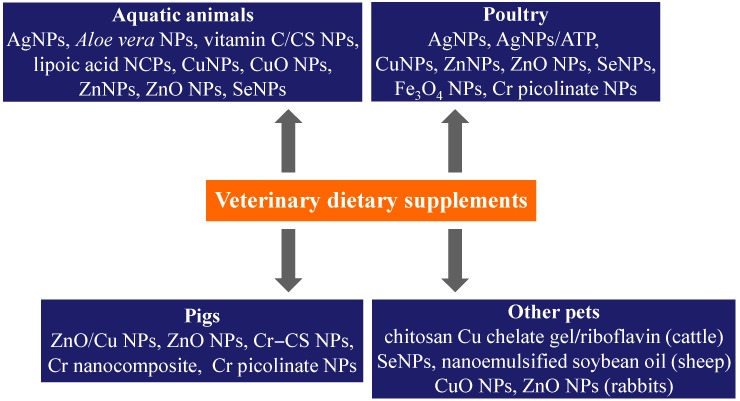
Summary of most frequently used veterinary dietary nanosupplements.

Nano-copper as a new growth promoter in the diet of growing New Zealand white rabbits was reported by Refaie et al. [294]. Similarly, the male five-week-old New Zealand white rabbits fed with the basal diet supplemented with 60 and 30 mg ZnO NPs /kg diet had higher body weight, daily weight gain, daily feed intake, serum total protein, globulin, IgG, and SOD compared to control animals and rabbits fed with a diet supplemented with bulk ZnO (60 mg/kg diet), suggesting that traditional zinc sources in rabbit diets could be replaced by 30 mg ZnO NP/kg diet [295]. On the other hand, Ismail and El-Araby [296] recommended the combined use of bulk and nanosized ZnO at ratio 1:1 in the dietary system of rabbit’s farms ensuring dietary supplementation with Zn as an essential element but reducing adverse effects such as lipid peroxidation and oxidant stress induced by the whole dose of ZnO NPs. A complete overview of the used nanoscale veterinary dietary supplements is shown in Figure 3.

## 6. Conclusions

The importance of balanced nutrition containing important nutrients, for example, vitamins or antioxidants, in sufficient amount needed for health of humans and animals is indisputable. Moreover, recently, physicians attribute an increasing significance to the consumption of food products containing special effective ingredients to prevent and improve the health of people suffering from certain diseases (e.g., cancer, diabetes, hyperlipidemia, mental disorders, osteoporosis, various malabsorption, etc.). The fortification of food products with such dietary supplements/nutraceuticals can be easily used in practice when an appropriate stability of the active ingredient in the formulation could be guaranteed at least until the date of consumption (expiration date). For these purposes, nanoformulations of active compounds prepared using biodegradable nature- or semisynthetic-based nanocarriers, such as polymeric matrices, micelles, liposomes, nanoemulsions, solid lipid NPs, nanostructured lipid carriers, or appropriate inorganic matrices are especially favorable, securing not only enhanced stability but also frequently controlled release of nutrients. Definitely, based on previous thorough experiments, for supplementation of food products (e.g., bread, butter, yogurt, cake, biscuit) or beverages (e.g., milk, juice) with individual healthy ingredients, the most convenient nanoformulation could be selected and used. Unlike nutraceuticals that are available for sale in pharmacy and could be overused by some careless consumers resulting in possible harmful side effects, at fortification of food products with dietary supplements/nutraceuticals, the excessive consumption of these compounds is excluded. However, even though nanoformulations enhance the bioavailability and increase the stability of individual active ingredients, all nanoscale materials applied in food industry should be used advisedly and only after in-depth investigation of cytotoxicity due to possible increased nanosize-based toxicity effects (e.g., surface reactivity of NPs), which could result in unspecified toxic effects also in humans or animals. Therefore, an increased attention should be paid to the influence of risk factors associated with their applications and possible adverse/hazardous effects to humans and animals, observing the relevant guidelines, regulations, and directives issued by the European Commission and the EFSA.

## Abbreviations

AChE (acetylcholinesterase); Alb (albumin); AST (astaxanthin); ATP (adenosine triphosphate); α-Lalb (α-lactalbumin); α-LLA (α-linoleic acid); α-LNA (α-linolenic acid); BAP (bioactive association platform); BMD (bone mineral densities); β-Car (β-carotene); β-Lglb (β-lactoglobulin); CDs (cyclodextrins); CPE (*Citrus reticulata* Blanco cv. unshiu peel extract); CrPic (chromium picolinate); CS (chitosan); CUR (curcumin); CVD (cardiovascular disease); CVPs (*Catathelasma ventricosum* polysaccharides); DESs (delivery systems); DHA (docosahexaenoic acid); DISs (dietary supplements); EE (encapsulation efficiency); EFSA (European Food Safety Authority); EGCG (epigallocatechin gallate); FA (folic acid); FDA (U.S. Food and Drug Administration); FSMPs (foods for special medical purposes); GH (growth hormone); GI (gastrointestinal); Glb (globulin); GPx (glutathione peroxidase); GSH (glutathione); HDL (high-density lipoprotein); LCT (long-chain triglycerides); LDHs (layered double hydroxides); LDL (low-density lipoprotein); MCT (medium chain triglycerides); MDA (malondialdehyde); Met (methionine); MPs (microparticles); MSPs (mesoporous particles); NE (nanoemulsion); NLCs (nanostructured lipid carriers); NPs (nanoparticles); NPES (nanopowdered eggshell); NRC (National Research Council); OVX (ovariectomized); PC (phosphatidylcholine); PGA (polyglutamic acid); PUFAs (polyunsaturated fatty acids); Q (quercetin); r-CMs (re-assembled casein micelles); RES (resveratrol); SCas (sodium caseinate); SOD (superoxide dismutase); SPI (sesame protein isolate); STZ (streptozotocin); TBFs (tartary buckwheat rich in flavonoids); ZLNPs (zein NPs encapsulating lutein).
